# RAB39B Deficiency Impairs Learning and Memory Partially Through Compromising Autophagy

**DOI:** 10.3389/fcell.2020.598622

**Published:** 2020-12-08

**Authors:** Mengxi Niu, Naizhen Zheng, Zijie Wang, Yue Gao, Xianghua Luo, Zhicai Chen, Xing Fu, Yanyan Wang, Ting Wang, Manqing Liu, Tingting Yao, Peijie Yao, Jian Meng, Yunqiang Zhou, Yunlong Ge, Zhanxiang Wang, Qilin Ma, Huaxi Xu, Yun-wu Zhang

**Affiliations:** ^1^Department of Neurology, The First Affiliated Hospital of Xiamen University, Xiamen, China; ^2^Fujian Provincial Key Laboratory of Neurodegenerative Disease and Aging Research, Institute of Neuroscience, School of Medicine, Xiamen University, Xiamen, China; ^3^Department of Neurosurgery, Xiang’an Hospital of Xiamen University, Xiamen, China; ^4^Department of Neurosurgery, The First Affiliated Hospital of Xiamen University, Xiamen, China

**Keywords:** autophagy, learning and memory, NMDA receptors, RAB39B, rapamycin

## Abstract

*RAB39B* is located on the X chromosome and encodes the RAB39B protein that belongs to the RAB family. Mutations in *RAB39B* are known to be associated with X-linked intellectual disability (XLID), Parkinson’s disease, and autism. However, the patho/physiological functions of RAB39B remain largely unknown. In the present study, we established *Rab39b* knockout (KO) mice, which exhibited overall normal birth rate and morphologies as wild type mice. However, *Rab39b* deficiency led to reduced anxiety and impaired learning and memory in 2 months old mice. Deletion of *Rab39b* resulted in impairments of synaptic structures and functions, with reductions in NMDA receptors in the postsynaptic density (PSD). RAB39B deficiency also compromised autophagic flux at basal level, which could be overridden by rapamycin-induced autophagy activation. Further, treatment with rapamycin partially rescued impaired memory and synaptic plasticity in *Rab39b* KO mice, without affecting the PSD distribution of NMDA receptors. Together, these results suggest that RAB39B plays an important role in regulating both autophagy and synapse formation, and that targeting autophagy may have potential for treating XLID caused by *RAB39B* loss-of-function mutations.

## Introduction

Intellectual disability (ID) refers to a group of neurodevelopmental disorders characterized by severe defects in both cognition (with an IQ score of less than 70) and adaptive behavior, which start before the age of 18 years (Vissers et al., 2016; Iwase et al., 2017). Although environmental factors such as maternal drug abuse during pregnancy and birth complications can cause ID, increasing evidence indicates that genetics also plays a significant role in the pathogenesis of ID (Vissers et al., 2016; Iwase et al., 2017). It is estimated that genomic/genetic variants contribute to about 25–50% of ID incidence. X-linked ID (XLID) resulting from genomic/genetic variants on X chromosome is one of the most common ID types and has a prevalence of 10–15% in the whole ID population (Peng et al., 2017). Because of the hemizygosity of X chromosomes in males, XLID occurs much more in males than females. So far genomic/genetic variants in more than 100 genes on X chromosome have been associated with XLID (Peng et al., 2017). However, detailed molecular pathways underlying ID pathogenesis driven by each of these genes remain largely unknown; and elucidation of underlying molecular mechanisms shall provide new insights into disease therapeutic strategies.

The *RAB39B* gene is located on X chromosome (Xq28) and encodes the RAB39B protein that belongs to the Rab protein family (Cheng et al., 2002). Similar to other small GTPases, the activity of Rab proteins is regulated by GTP-bound active and GDP-bound inactive states. As an evolutionarily conserved vesicle transporter regulator group, Rab proteins localize to different membrane structures to regulate vesicle trafficking (Corbeel and Freson, 2008). Recent studies have found that loss-of-function *RAB39B* mutations are associated with various diseases including XLID, autistic spectrum disorder (ASD), and Parkinson’s disease (PD) (Supplementary Table 1; Giannandrea et al., 2010; Mata et al., 2015; Guldner et al., 2016; Lesage et al., 2016; Shi et al., 2016; Ciammola et al., 2017; Woodbury-Smith et al., 2017; Santoro et al., 2020). However, the physiological functions of RAB39B as well as its pathological roles in disease pathogenesis remain largely elusive.

Several reports suggested that RAB39B may regulate protein trafficking and the PI3K-AKT-mTOR pathway. On one hand, one study found that RAB39B could interact with PICK1 to control trafficking of the AMPA receptor subunit GluA2 from the endoplasmic reticulum to the Golgi and then to cell surface; downregulation of RAB39B skewed AMPA receptor composition toward non-GluA2-containing calcium-permeable forms and hence altered synaptic activity of mouse hippocampal neurons (Mignogna et al., 2015). Consistently, downregulation of RAB39B in neurons altered the number and morphology of neurite growth cones and reduced presynaptic buttons, suggesting a role of RAB39B in synapse formation and maintenance (Giannandrea et al., 2010). On the other hand, RAB39B was found to interact with PI3K components and loss of RAB39B could promote PI3K-AKT-mTOR activity, resulting in altered cortical neurogenesis, macrocephaly, and ASD-like behaviors in mice (Zhang et al., 2020). Nevertheless, whether loss of RAB39B lead to various disease phenotypes through the same mechanism has yet to be determined.

## Materials and Methods

### Animals

*Rab39b* KO mice were generated as described in Supplementary Materials and Methods. C57BL/6J wild type (WT) mice were provided by Xiamen University Laboratory Animal Center. All animal procedures were approved by the Animal Ethics Committee of Xiamen University and were conducted in accordance with the National Institutes of Health Guidelines for the Care and Use of Laboratory Animals. The mice used were all 2 months old, except otherwise indicated specifically.

### Antibodies, Reagents, and Western Blot

Antibodies against RAB39B (Cat# D-12162-1-AP), GluA2 (Cat# 11994-1-AP), GluN2A (Cat# 19953-1-AP), GluN2B (Cat# 19954-1-AP), and tyrosine hydroxylase (TH, Cat# 66334-1-IG) were from Proteintech. Antibodies against GFAP (Cat# 3670s), β-actin (Cat# 8457s), p62 (Cat# 5114s), GluN1 (Cat# 5704s), PSD95 (Cat# 3450s), LC3B (Cat# 3868s), S6 (Cat# 2217s), and phosphorylated S6 (Ser240/244) (Cat# 5364s) were from Cell Signaling Technology. Antibodies against GluA1 (Cat# 04-855), GluA3 (Cat# MAB5416), and SYN1 (Cat# AB1543) were from Millipore. Antibodies against NeuN (Cat# ab177487), Iba1 (Cat# 016-20001), and synaptophysin (SYP) (Cat# S5768) were from Abcam, Wako, and Sigma-Aldrich, respectively. Goat anti-rabbit (Cat# 31460) or anti-mouse (Cat# 31430) IgG (HCL) secondary antibodies conjugated with horseradish peroxidase were from Thermo Fisher Scientific.

The mTOR inhibitor rapamycin (Cat# HY-10219) was from MedChemExpress. DMSO (Cat# 20688) was from Thermo Fisher Scientific. 4′,6-diamidino-2-phenylindole (DAPI) (Cat# D9542) was from Sigma-Aldrich. Complete Protease Inhibitor and PhosSTOP Cocktails were from Roche.

Different protein levels in mouse tissues and cells were determined by western blot. Detailed procedures are presented in Supplementary Materials and Methods. Protein band intensity was quantified by densitometry using the Image J software (National Institutes of Health).

### Cell Culture

Mouse neuroblastoma N2a cells were maintained in high glucose DMEM (Hyclone) supplemented with 10% (vol/vol) fetal bovine serum (FBS, Gibco), 100 units/ml penicillin (Gibco), and 100 μg/ml streptomycin (Gibco), and incubated at 37°C in humidified air with 5% CO_2_.

### RNA Interference

An siRNA sequence (sense: 5′-UCAUUCUUCAGAAGAGGUU TT-3′; antisense: 5′-AACCUCUUCUGAAGAAUGATT-3′) targeting mouse *Rab39b* was designed and synthesized by GenePharma. A scrambled siRNA sequence (sense: 5′-UUCUC CGAACGUGUCACGUTT-3′; antisense: 5′-ACGUGACACGUU CGGAGAATT-3′) was used as a negative control. These siRNAs were labeled with the fluorescent dye Cy5 and were transfected into N2a cells using Lipofectamine 3000 Reagent (Invitrogen), following the manufacturer’s protocol.

### DNA Construct and Transfection

The RFP-GFP-LC3B vector was from Biovector Science Lab, Inc. The construct was transfected into N2a cells by Turbofect transfection reagent (Thermo Fisher Scientific), following the manufacturer’s protocol.

### Quantitative Real Time-PCR (qRT-PCR)

Total RNAs were extracted using the TRIzol Reagent (Invitrogen), and reverse-transcribed using the ReverTra Ace qPCR RT Kit (Toyobo), following the manufacturers’ instructions. Equal amounts of cDNA from each sample were subjected to qRT-PCR. Primers used were as follows:

For *Rab39b*:

forward primer: 5′-CTGGGATACAGCGGGTCAAG-3′;

reverse primer: 5′-GAAGGACCTGCGGTTGGTAA-3′;

For β*-actin*:

forward primer: 5′-AGCCATGTACGTAGCCATCCA-3′;

reverse primer: 5′-TCTCCGGAGTCCATCACAATG-3′.

### Immunofluorescence

Mouse brain sections were treated with citrate buffer (pH 7.0) for 10 min for antigen retrieval, permeabilized, and blocked in PBS containing 0.5% (vol/vol) Triton X-100 and 10% (vol/vol) normal goat serum at room temperature for 1 h. After washing with PBS for three times, samples were incubated with primary antibodies in blocking buffer overnight at 4°C, and then with fluorescence-conjugated secondary antibodies in blocking buffer at room temperature for 1 h. After PBS washing, samples were stained with DAPI for 10 min. Images were acquired using a confocal fluorescence microscope (Nikon).

Cells transfected with *Rab39b* siRNA and RFP-GFP-LC3B were fixed in 4% (wt/vol) paraformaldehyde for 15 min. After washing with PBS, cells were stained with DAPI for 10 min and visualized under a confocal fluorescence microscope (Nikon). The fluorescence intensity was quantified by densitometry using Image J.

### Mouse Behavioral Tests

Detailed procedures for behavioral experiments are presented in Supplementary Materials and Methods.

### Electrophysiology

Electrophysiological recordings were performed as previously described (Wen et al., 2010, 2011; Zeng et al., 2019). More details are provided in Supplementary Materials and Methods.

### Golgi Staining

Freshly dissected mouse brains were subjected to Golgi staining using the FD Rapid Golgi Stain system (FD Neuro Technologies), following the manufacturer’s instructions. Brains were sliced with a vibratome (Leica) at a thickness of 100 μm. After dehydration, Golgi-stained neurons and spines were visualized under a confocal microscope (Nikon). Apical distal spine density of neurons in cortical V1/V2 and hippocampal CA1 regions were quantified by densitometry using Image J.

### Electron Microscope Analysis

Synapse structures were assayed using a transmission electron microscope (Ung et al., 2018; Zhao D. et al., 2019). More details are provided in Supplementary Materials and Methods.

### Preparation of Synaptosomal and PSD Fractions

PSD fractions from mouse hippocampus were dissected as previously described (Wang et al., 2013). More details are provided in Supplementary Materials and Methods.

### Rapamycin Treatment

Rapamycin treatment procedure was based on a previously reported study (Zhou et al., 2009). Briefly, rapamycin was first dissolved in DMSO (100 mg/ml) and then diluted with 0.9% (wt/vol) saline to 1 mg/ml. DMSO vehicle were dissolved in 0.9% saline to a concentration of 1% (vol/vol). 2 months old *Rab39b* KO mice were intraperitoneally injected with rapamycin at 7.5 mg/kg or DMSO vehicle at 7.5 ml/kg per day for 7 consecutive days. Starting from the 8th day, mice were subjected to electrophysiological analysis or behavioral tests, during which processes mice were injected with rapamycin continually. On the 14th day, mice were sacrificed for subsequent biochemical analysis.

### Statistics

Data represent mean ± standard error of means (SEM). Statistical analysis was performed using Graphpad Prism 6 or SPSS 13.0 softwares. Detailed statistical data and methods for each comparison are indicated in the text and/or in Supplementary Table 2.

## Results

### Generation and Characterization of *Rab39b* Knockout (KO) Mice

RAB39B was previously reported as a neural-specific protein (Giannandrea et al., 2010). Here we also confirmed that RAB39B was specifically expressed in the brain but not other tissues detected in 2 months old C57BL/6J mice (Supplementary Figure 1A). The expression level of RAB39B was comparable in different brain regions including cortex, hippocampus, cerebellum, and midbrain (Supplementary Figure 1B). Moreover, we found that mouse RAB39B was predominantly expressed in primary neurons, with minimal detection in primary astrocytes and no detection in primary microglia (Supplementary Figure 1C). At early postnatal stages, the expression of mouse RAB39B in the brain was gradually increased and showed a pattern correlated well to those of neuronal proteins GluN1 and synapsin 1 (Supplementary Figure 1D).

**FIGURE 1:**
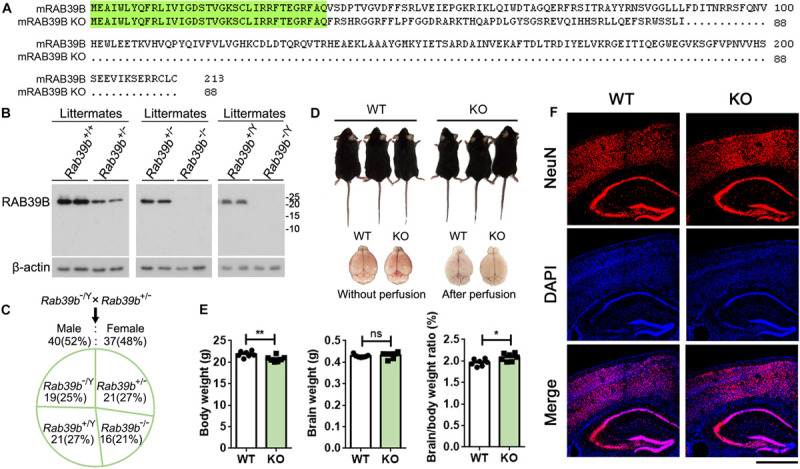
Characterization of *Rab39b* KO mice. **(A)** Sequence alignment of RAB39B proteins of wild type (213 amino acids) and knockout (KO, 88 amino acids) mice. Conserved residues are highlighted in green. **(B)** RAB39B proteins in brain samples of wild type (*Rab39b*^+/+^ and *Rab39b*^+/Y^), heterozygous female (*Rab39b*^+/–^), homozygous female (*Rab39b*^– /–^), and hemizygous male (*Rab39b*^– /Y^) KO mice from same litters were detected by western blot. **(C)** *Rab39b*^+/–^ female and *Rab39b*^– /Y^ male mice were crossed. Numbers of males and females, as well as different genotypes of the offspring were counted for studying if they follow a Mendelian frequency. **(D)** Observation of the overall body and brain (before and after perfusion) morphologies of *Rab39b* KO mice and their wild type (WT) littermate controls. **(E)** Comparison of body weight, brain weight, and the ratio of brain/body weight between *Rab39b* KO mice and their WT littermate controls at 2 months of age. Data represent mean ± SEM, *n* = 7 for each group, ns: not significant, *p < 0.05, **p < 0.01, Mann-Whitney test. **(F)** Cortical and hippocampal regions of WT and *Rab39b* KO mice at 2 months of age were immunostained with an anti-NeuN antibody (red) and stained with DAPI (blue), and observed under a confocal microscope. Scale bar, 500 μm.

RAB39B is highly conserved between mouse and human, with only one amino acid difference (Supplementary Figure 2), suggesting their functional conservation and that outcome from mouse study may predict authentic functions of human RAB39B. Therefore, to study the physiological function of RAB39B *in vivo*, we used the transcription activator-like effector nucleases (TALEN) technique to generate *Rab39b* KO mice in a C57BL/6J background and obtained a mouse line that had a two nucleotide “GT” deletion within the *Rab39b* protein coding sequence (CDS sites 106–107) (Supplementary Figures 3A–D). Such a deletion resulted in a frame shift and early truncation of RAB39B (Figure 1A). RAB39B was undetectable in either hemizygous *Rab39b* KO male mice or homozygous *Rab39b* KO female mice (Figure 1B), indicating the loss of wild type RAB39B in this mouse line. Mutant *Rab39b* mRNA levels was also decreased compared to WT *Rab39b* mRNA levels (Supplementary Figure 3E). In addition, RAB39B protein levels were relatively low in heterozygous *Rab39b* KO female mice compared to WT littermate female mice (Figure 1B).

Both *Rab39b* KO male and female mice were viable and fertile. When heterozygous *Rab39b* KO female mice were crossed with hemizygous *Rab39b* KO male mice, the genotypes (chi-squared test, *p* = 0.83) as well as the sex ratio (chi-squared test, *p* = 0.73) of the offspring followed a Mendelian frequency (Figure 1C).

Because *RAB39B* is an X-linked gene with a recessive inheritance and X-linked ID is more common in males, we focused our study on male mice and unless specific mentioning, mice used throughout the study refer to males only. The overall body and brain morphologies of *Rab39b* KO mice were indistinguishable from those of littermate controls at 2 months of age (Figure 1D). The body weight but not the brain weight of *Rab39b* KO mice were lighter than those of littermate controls at 2 months of age, resulting in an increased brain/body weight ratio in *Rab39b* KO mice (Figure 1E). Moreover, we found no obvious neuronal abundance differences in cortical and hippocampal regions between *Rab39b* KO and WT mice at 2 months of age (Figure 1F and Supplementary Figure 3F). These results suggest that RAB39B is not essential for normal development in mice.

### Altered Behavioral Phenotypes in *Rab39b* KO Mice

**FIGURE 2:**
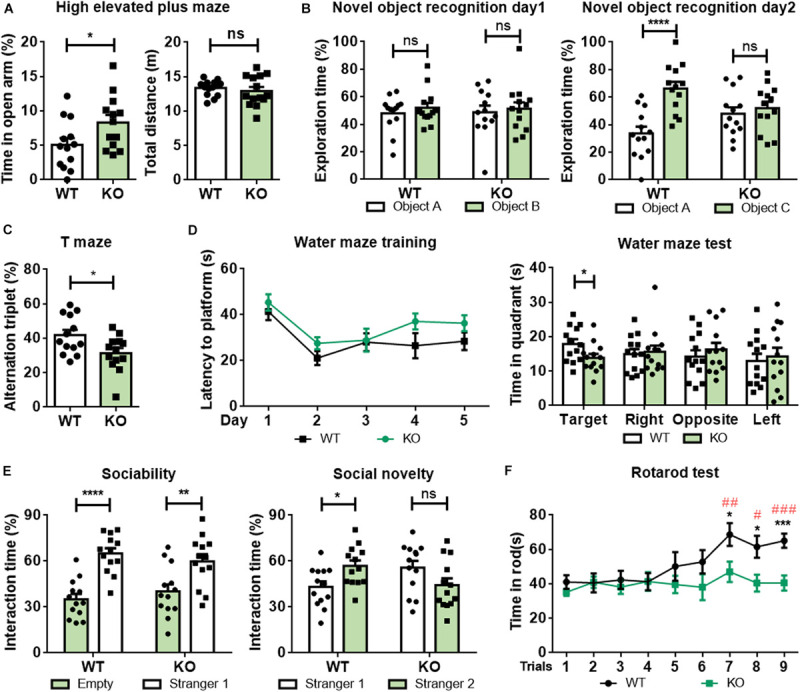
*Rab39b* KO mice exhibit altered behavioral phenotypes. Two months old *Rab39b* KO mice and WT littermate control mice were subjected to various behavioral tests. **(A)** Mice were studied for their time staying in the open arm and total travel distance in high elevated plus maze tests. **(B)** In novel object recognition tests, mice were evaluated for their recognition of two identical objects A and B on day 1 and identification of a novel object C on day 2. **(C)** Mice were tested for their spontaneous alternation in T maze tests. **(D)** Mice were analyzed for their escape latency in Morris Water Maze tests within a 5 day training period. On the 6th day, mice were assayed for time spent in the target and the other three quadrants. **(E)** In three-chamber social interaction tests, mouse sociability was studied by comparing their interactions with a stranger mouse (stranger 1) and with an empty cage. Social novelty was studied by comparing their interactions with the familiar mouse (stranger 1) and with a new stranger mouse (stranger 2). **(F)** Mice were tested for their time staying on a rotarod three times a day for three consecutive days. Data represent mean ± SEM, *n* = 13 for each group. For comparisons between WT and KO in **(A–F)**, ns: not significant, *p < 0.05, **p < 0.01, ***p < 0.001, ****p < 0.0001, Mann-Whitney test. For comparisons between rotarod staying times of other trials and rotarod staying time of the first trial within WT in **(F)**, ^#^*p* < 0.05, ^##^*p* < 0.01, ^###^*p* < 0.001, Mann-Whitney test.

In open field tests, *Rab39b* KO mice showed no differences in moving speed, the time spent in the center, and total travel distance compared to WT littermates (Supplementary Figure 4A). However, in high elevated plus maze tests, *Rab39b* KO mice spent more time in the open arms than WT littermates (Figure 2A), implying that loss of *Rab39b* reduced mouse anxiety.

Since loss of function mutations in *RAB39B* cause ID, autism, and PD symptoms in humans, we next studied whether loss of *Rab39b* affects mouse behaviors resembling these diseases. In novel object recognition tests, both *Rab39b* KO and WT mice spent similar time exploring the two identical objects during the training (Figure 2B, left panel). However, although WT mice spent more time exploring the novel object than the familiar object, *Rab39b* KO mice explored the novel and the familiar objects with similar time (Figure 2B, right panel), suggesting that loss of *Rab39b* impaired recognition memory. Moreover, *Rab39b* KO mice showed reduced spontaneous alternations compared to WT mice in T-maze tests (Figure 2C), indicating that loss of *Rab39b* also impaired short-term working memory. During Morris water maze tests, WT and *Rab39b* KO mice had similar total travel distance and swimming speed (Supplementary Figure 4B). However, despite exhibiting little difference from WT controls in their escape latency to the hidden platform during the training (Figure 2D, left panel), *Rab39b* KO mice spent significantly less time in the target quadrant than WT controls during the testing (Figure 2D, right panel), suggesting impaired spatial memory in *Rab39b* KO mice.

In three-chamber social interaction tests, we found that both *Rab39* KO mice and WT mice spent significantly more time approaching the cage with a strange mouse (Stranger 1) than the empty cage (Figure 2E, left panel). However, when another strange mouse (Stranger 2) was placed into the empty cage, WT mice preferred to explore Stranger 2, whereas *Rab39b* KO mice showed no such a preference (Figure 2E, right panel). These results suggest that *Rab39b* deficiency mainly impairs social novelty recognition rather than sociability.

In rotarod tests, mice were given three tests per day for 3 consecutive days. WT and *Rab39b* KO mice stayed on the rod for similar time periods during the first four tests. But then WT mice had increased latency for staying on the rod in following tests, whereas *Rab39b* KO mice failed to improve the latency (Figure 2F). In four-limb hanging tests which assess rodents’ muscle strength, *Rab39b* KO mice showed similar ability in hanging time and hanging impulse compared to WT mice (Supplementary Figure 4C). These results suggest that loss of *Rab39b* compromises motor skill learning but does not affect motor ability at a young age.

### *Rab39b* KO Mice Exhibit Defects in Synaptic Function and Structure and PSD Composition

**FIGURE 3:**
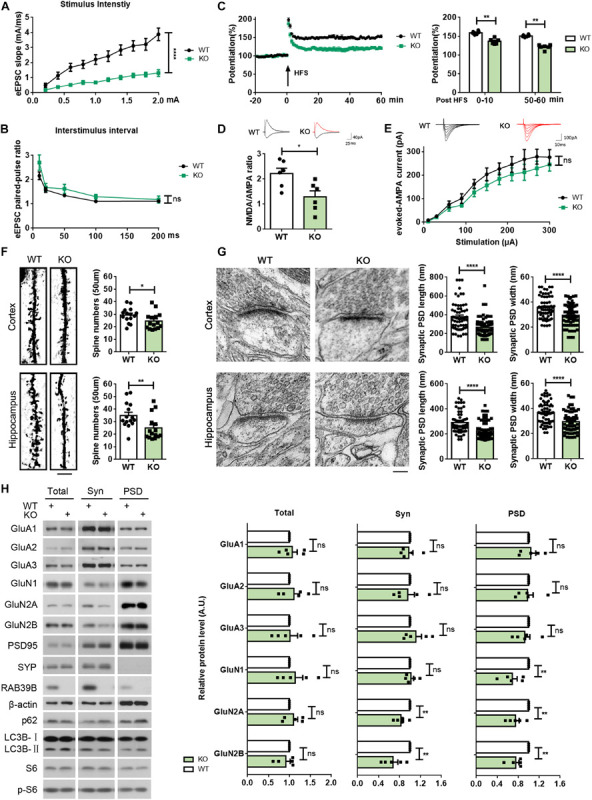
*Rab39b* KO mice exhibit defects in synaptic function and structure and PSD composition. **(A)** Changes of eEPSCs were recorded in the CA1 region when increased stimulations were given in the CA3 region of WT and *Rab39b* KO mice. Input–output curves were subjected to comparison. Data represent mean ± SEM, *n* = 6 slices from four mice per group, ****p < 0.0001, repeated measures ANOVA. **(B)** Paired-pulse ratios of eEPSCs in the CA1 pyramidal neurons were subjected to comparison. Data represent mean ± SEM, *n* = 6 slices from four mice per group, ns: not significant, one-way ANOVA followed with Dunnett’s test. **(C)** LTP was induced by a two-train (100 Hz, 30 s interval) high frequency stimulation (HFS) in the CA3 region. The left panel shows representative fEPSP recording traces from 20 before to 60 min after HFS in the CA1 region. The right panel shows comparisons of mean potentiation from the fEPSP slopes calculated between 0–10 and 50–60 min after HFS. Data represent mean ± SEM, *n* = 6 slices from four mice per group, **p < 0.01, Mann-Whitney test. **(D)** AMPA receptor- and NMDA receptor-mediated EPSCs were recorded from same hippocampal CA1 neurons and NMDA/AMPA receptor response ratios were calculated for comparison.Data represent mean ± SEM, *n* = 6 cells per group, *p < 0.05, Mann-Whitney test. **(E)** AMPA-eEPSCs were recorded in hippocampal CA1 neurons at different stimulus intensities for comparison. Data represent mean ± SEM, *n* = 7 cells per group, ns: not significant, repeated measures ANOVA. **(F)** Cortical and hippocampal regions of WT and *Rab39b* KO mice were subjected to Golgi staining and microscopy. Scale bar, 5 μm. Apical distal spine numbers of neurons in cortical V1/V2 and hippocampal CA1 regions were quantified, respectively, for comparison. Data represent mean ± SEM, *n* = 15 neurons from three mice per group, *p < 0.05, **p < 0.01, Mann-Whitney test. **(G)** Cortical V1/V2 and hippocampal CA1 regions of WT and *Rab39b* KO mice were subjected to electron microscopy. Scale bar, 100 nm. PSD length and width were quantified for comparison. Data represent mean ± SEM, *n* = 66 synapses from three mice per group, ****p < 0.0001, two-tailed Student’s *t*-test. **(H)** Equal quantities of brain samples of WT and *Rab39b* KO mice were fractionated to acquire total lysates, synaptosomal (Syn) fractions, and PSD fractions. Samples were subjected to western blot to detect the proteins indicated. Levels of proteins indicated were quantified by densitometry, normalized to those of β-actin, and compared to respective WT controls (set to one arbitrary units, A.U.). Data represent mean ± SEM, *n* = 5 for each group, ns: not significant, **p < 0.01, Mann-Whitney test.

Since impaired learning and memory is associated with altered synaptic plasticity (Acquarone et al., 2019; Zhao D. et al., 2019), we carried out electrophysiological study on synaptic functions. When evoked excitatory postsynaptic currents (eEPSCs) in the hippocampal CA1 stratum radiatum were recorded by stimulating the Schaffer collateral (SC)/commissural pathway at various intensities, a marked reduction in eEPSC slopes in *Rab39b* KO mice was detected compared to WT mice (Figure 3A). Since the ratio of paired-pulse facilitation was not significantly different between *Rab39b* KO and WT mice (Figure 3B), the reduced eEPSCs in *Rab39b* KO mice are probably caused by post-synaptic defects. LTP at the SC-CA1 region was also dramatically reduced in *Rab39b* KO mice compared to WT mice (Figure 3C).

AMPA receptors and NMDA receptors play crucial roles in synaptic function and memory formation (Scannevin and Huganir, 2000; Hettinger et al., 2018; Hanada, 2020). To characterize potential differences of the two receptors in *Rab39b* KO mice, we measured NMDA/AMPA receptor response ratios and evoked AMPA excitatory postsynaptic currents (AMPA-eEPSCs) in hippocampal CA1 neurons of WT and *Rab39b* KO mice. We found that NMDA/AMPA receptor response ratios were significantly decreased in *Rab39b* KO neurons compared to WT controls (Figure 3D), whereas AMPA-eEPSC amplitudes were not significantly altered upon loss of *Rab39b* (Figure 3E). These results suggest that NMDA receptor function rather than AMPA receptor function is impaired in *Rab39b* KO mice.

We next evaluated and found significant spine density reductions in both cortical and hippocampal neurons of *Rab39b* KO mice when compared to their littermate controls (Figure 3F). We also performed an ultra-structure analysis of post-synaptic density (PSD) by transmission electron microscopy and found that both PSD length and width in the cortex and hippocampal CA1 regions of *Rab39b* KO mice were significantly reduced compared to WT controls (Figure 3G), whereas synaptic vesicle numbers in both regions were not different (Supplementary Figure 5A).

We next studied and found that the total amounts of AMPA and NMDA receptor subunits showed no differences between *Rab39b* KO and WT mice (Figure 3H and Supplementary Figure 5B). However, the PSD fraction distribution of NMDA receptors including GluN1, GluN2A, and GluN2B were markedly reduced in *Rab39b* KO mice (Figure 3H), whereas the PSD distribution of AMPA receptors including GluA1, GluA2, and GluA3 remained unchanged (Figure 3H). These findings are consistent with the specific NMDA receptor function impairment in *Rab39b* KO mice as revealed by electrophysiological studies (Figures 3D,E). Together, these results indicate that RAB39B plays an important role in maintaining synaptic plasticity and structure and PSD composition.

### Loss of *Rab39b* Impairs Autophagy

**FIGURE 4:**
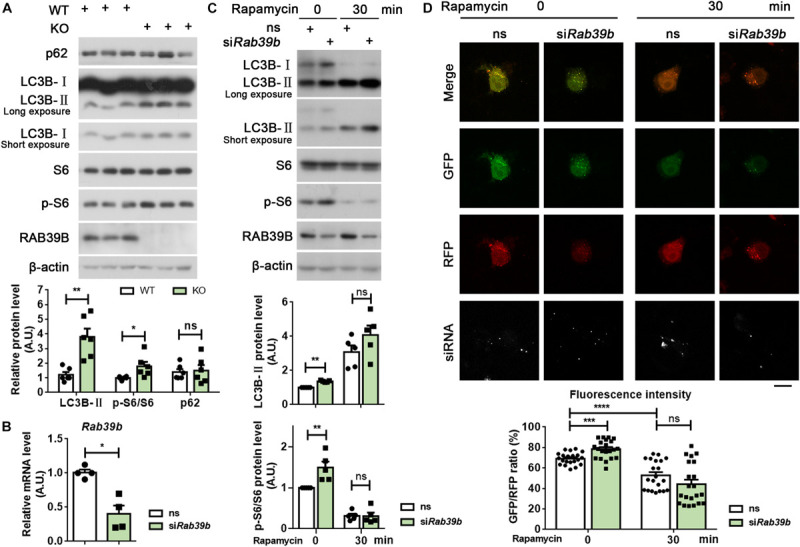
RAB39B deficiency impairs autophagy. **(A)** Equal protein quantities of hippocampus lysates derived from WT and *Rab39b* KO mice were subjected to western blot for the proteins indicated. Levels of p62, LC3B-II, and S6 phosphorylated at S240/244 (p-S6) were quantified by densitometry and normalized to those of β-actin or S6, respectively, and compared to WT controls (set to one arbitrary units, A.U.). Data represent mean ± SEM, *n* = 6 for each group, ns: not significant, *p < 0.05, **p < 0.01, Mann-Whitney test. **(B)** N2a cells were transfected with scrambled siRNA (ns) or an siRNA targeting *Rab39b* (si*Rab39b*) for 60 h. After RNA extraction and reverse transcription, *Rab39b* mRNA levels were detected by qRT-PCR, normalized to those of β-actin, and compared to ns controls (set to one A.U.). Data represent mean ± SEM, *n* = 4 for each group, *p < 0.05, Mann-Whitney test. **(C)** N2a cells were transfected with ns or *siRab39b* for 60 h. Cells were then treated with 250 nM rapamycin for 0 or 30 min. Cells lysates were subjected to western blot for the proteins indicated. LC3B-II and p-S6 levels were quantified for comparison to respective ns controls (set to one A.U.). Data represent mean ± SEM, *n* = 5 for each group, ns: not significant, **p < 0.01, Mann-Whitney test. **(D)** N2a cells were first transfected with ns or *siRab39b* for 36 h and then transfected with RFP-GFP-LC3B. After another 24 h, cells were treated with 250 nM rapamycin for 0 or 30 min. Images were acquired by confocal microscopy. Red and green colors indicate RFP and GFP, respectively. The siRNA was depicted in white. Scale bar, 10 μm. Data represent mean ± SEM, *n* = 21 from three independent experiments for each group, ns: not significant, ***p < 0.001, ****p < 0.0001, two-tailed Student’s *t*-test.

One recent study found that RAB39B regulates the PI3K-AKT-mTOR signaling (Zhang et al., 2020). Here we also found that phosphorylated S6 levels were increased in the hippocampus, cortex, and midbrain of *Rab39b* KO mice compared to WT controls (Figure 4A and Supplementary Figure 6), corroborating an upregulation of the PI3K-AKT-mTOR signaling upon loss of RAB39B. Since the PI3K-AKT-mTOR pathway inhibits autophagy, we further studied whether RAB39B regulates autophagy. Levels of LC3B-II, an autophagy marker were increased in *Rab39b* KO mice (Figure 4A and Supplementary Figure 6). Consistently, downregulation of RAB39B in mouse N2a cells resulted in increased levels of phosphorylated S6 and LC3B-II (Figures 4B,C). However, rapamycin treatment induced LC3B-II increase and phosphorylated S6 decrease in both control and RAB39B-downregulated cells, with comparable levels (Figure 4C). Although an increase of LC3B-II levels usually suggests autophagy activation, LC3B-II level increase may also be caused by a deficiency in the autophagosome–lysosome fusion that blocks autophagic flux and thus LC3B-II degradation in the autophagolysosome. Therefore, we downregulated RAB39B in N2a cells and then transfected them with an RFP-GFP-LC3B construct. The advantage of using this RFP-GFP-LC3B construct is that the GFP signal but not the RFP signal is quenched in the acidic and degradative autophagolysosome, so that a change of the GFP/RFP ratio indicates a change of the autophagolysosome formation (Kimura et al., 2007). Herein, we found that the GFP/RFP signal ratio was increased in RAB39B-downregulated cells compared to controls (Figure 4D), suggesting that RAB39B deficiency caused a reduction in autophagolysosome formation and thus a decreased autophagic flux; and this is consistent with the elevated PI3K-AKT-mTOR signaling. However, when these cells were treated with rapamycin, the ratio of GFP/RFP signal in both control and RAB39B-downregulated cells were similarly reduced (Figure 4D). Together, these results indicate that RAB39B deficiency impairs autophagic flux at basal level but does not affect cellular response to autophagy stimulation.

### Rapamycin Treatment Partially Rescues Memory and LTP Defects in *Rab39b* KO Mice

**FIGURE 5:**
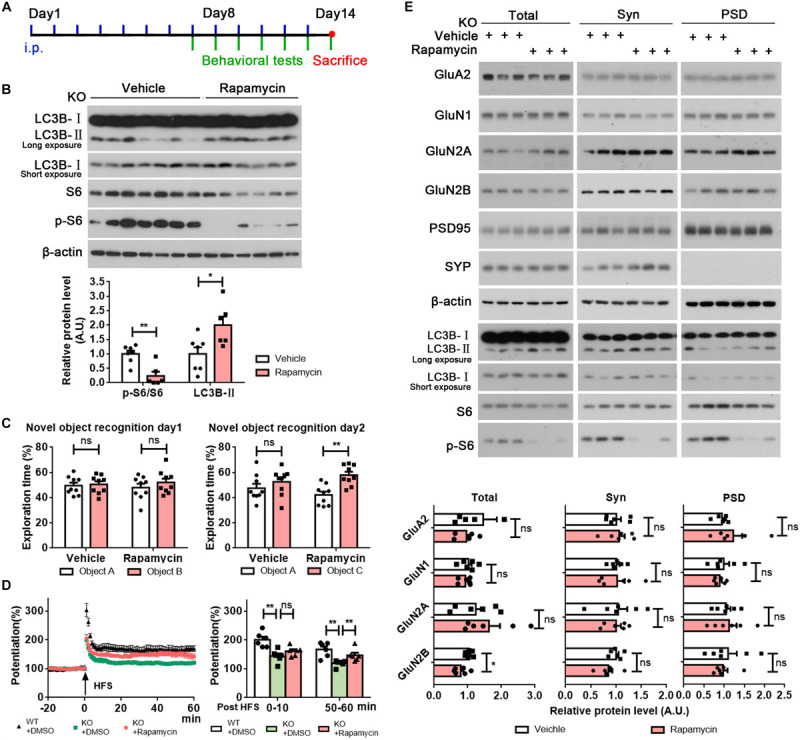
Rapamycin treatment partially rescues memory and LTP defects in *Rab39b* KO mice. **(A)** The workflow for rapamycin treatment and subsequent analysis. **(B)** Equal protein quantities of hippocampus lysates derived from *Rab39b* KO mice treated with rapamycin or vehicle were subjected to western blot for the proteins indicated. LC3B-II and phosphorylated S6 (p-S6) levels were quantified by densitometry, normalized to those of β-actin and S6, respectively, and compared to controls (set to one arbitrary units, A.U.). Data represent mean ± SEM, *n* = 7 for vehicle group, *n* = 6 for rapamycin group, *p < 0.05, **p < 0.01, Mann-Whitney test. **(C)** Treated *Rab39b* KO mice were subjected to novel object recognition tests to study their recognition of two identical objects A and B on day 1 and recognization of a novel object C on day 2. Data represent mean ± SEM, *n* = 9 for each group, ns: not significant, **p < 0.01, Mann-Whitney test. **(D)** LTP was induced by a two-train (100 Hz, 30 s interval) high frequency stimulation (HFS) in the CA3 region of mice. The left panel is representative fEPSP recording traces from 20 min before to 60 min after HFS in the CA1 region. The right panel shows comparisons of mean potentiation from the fEPSP slopes calculated between 0 and 10 and 50–60 min after HFS. Data represent mean ± SEM, *n* = 8 slices from four mice per group, ns: not significant, **p < 0.01, Mann-Whitney test. **(E)** Equal quantities of brain samples of treated *Rab39b* KO mice were fractionated to acquire total lysates, synaptosomal (Syn) fractions, and PSD fractions. Samples were subjected to western blot to detect the proteins indicated. GluN1, GluN2A, and GluN2B levels were quantified by densitometry, normalized to those of β-actin, and compared to respective controls (set to one A.U.). Data represent mean ± SEM, *n* = 6 for each group, ns: not significant, *p < 0.05, Mann-Whitney test.

To investigate whether promoting autophagy could rescue impaired memory and defective synaptic plasticity in *Rab39b* KO mice, we treated mice with rapamycin or DMSO vehicle by intraperitoneal injection (Figure 5A). We found that phosphorylated S6 levels were decreased and LC3B-II levels were increased in the hippocampal tissues of *Rab39b* KO mice treated with rapamycin when compared to those treated with vehicle (Figure 5B), indicating that autophagy was induced in mouse hippocampus by rapamycin treatment. Importantly, *Rab39b* KO mice treated with rapamycin showed significantly improved memory in novel object recognition tests when compared to controls (Figure 5C), while rapamycin treatment had no effect on affecting their locomotor activity in open field tests (Supplementary Figure 7A) or reversing the compromised short-term working memory in T maze tests (Supplementary Figure 7B), and the decreased anxiety in high elevated plus maze tests (Supplementary Figure 7C). Moreover, rapamycin treatment significantly improved impaired LTP in *Rab39b* KO mice (Figure 5D). However, the PSD distribution of NMDA receptor subunits (GluN1, GluN2A, and GluN2B) was not affected by rapamycin treatment (Figure 5E).

## Discussion

Gao et al. (2020) recently generated *Rab39b* KO mice (in C57BL/6J background) using the CRISPR/Cas9 technique. By comparing *Rab39b* KO and WT mice, they found that the expression of mouse RAB39B was high in the cortex, hippocampus, and substantia nigra, and that RAB39B was abundant in cortical and hippocampal neurons, as well as in dopaminergic neurons in the SNpc (Gao et al., 2020). Herein, we found that RAB39B was specifically expressed in neurons and comparably expressed in mouse hippocampus, cortex, midbrain, and cerebellum. Levels of Iba1 (a microglia marker) and GFAP (an astrocyte marker) in these brain regions were comparable between 2 months old WT and *Rab39b* KO mice (Supplementary Figures 5B, 8A), suggesting that loss of *Rab39b* has no effect on glial activation at this age. Since loss-of-function *RAB39B* mutations are associated with PD that has dramatic dopaminergic neuronal loss and dopamine dyshomeostasis during neurodegeneration (Masato et al., 2019; Shao and Le, 2019), we studied levels of dopamine and the dopaminergic neuronal marker tyrosine hydroxylase (TH) in the midbrain of 2 months old *Rab39b* KO mice. Interestingly, we found that TH levels were increased and dopamine levels were decreased in *Rab39b* KO mouse midbrain compared to controls (Supplementary Figures 8A,B). One possible reason for this is that loss of *Rab39b* leads to dopamine level reduction, which causes a compensatory increase in dopaminergic neuron numbers. How RAB39B deficiency exactly affects dopaminergic neurons at this age and whether the effect changes during aging deserve further scrutiny.

In another recent study, Zhang et al. (2020) also generated *Rab39b* KO mice (in C57BL/6N background) using the CRISPR/Cas9 technique and described that these mice had cortical neurogenesis impairment, macrocephaly, and social memory and motor skill learning deficits reminiscent patient phenotypes. While our current study confirmed phenotypic effect of *Rab39b* KO on motor skill learning and social memory, more importantly, we further investigated the detailed role of RAB39B in learning and memory. We found that *Rab39b* KO mice exhibited defects in short-term working memory, novel objection recognition memory, and spatial memory. Moreover, *Rab39b* KO mice exhibited impaired synaptic plasticity and altered synaptic structure and PSD composition. Since cognitive impairment is a critical feature of ID as a formal diagnosis of ID is made only when the IQ is scored to be less than 70 (Vissers et al., 2016), our results strengthen the causal effect of *RAB39B* mutations in the etiology of XLID. The PI3K-AKT-mTOR pathway is an important biological process that regulates cell differentiation, proliferation, migration, and metabolism during development. Dysfunction of this pathway may cause various neurological disorders such as ASD, focal cortical dysplasia, etc. (Costa-Mattioli and Monteggia, 2013; Dibble and Cantley, 2015). Zhang et al. (2020) found that RAB39B could interact with PI3K components and loss of RAB39B promoted the PI3K-AKT-mTOR pathway, leading to neural progenitor cell (NPC) over-proliferation and macrocephaly in mice at an age of postnatal day 20. Similarly, we also found that RAB39B deficiency resulted in markedly increased levels of phosphorylated S6, indicating the activation of the PI3K-AKT-mTOR pathway. However, we did not observe obvious macrocephaly in our *Rab39b* KO mice at 2 months of age. This discrepancy may be attributed to differences between mouse genetic backgrounds in the current study (C57BL/6J) and in Zhang et al. (2020) (C57BL/6N). C57BL/6J but not C57BL/6N mice carry a loss-of-function mutation in the nicotinamide nucleotide transhydrogenase gene and this makes C57BL/6J mice more susceptible to diet-induced obesity than C57BL/6N mice (Nicholson et al., 2010). Therefore, different metabolism and possibly other different biological processes between the two mouse strains may interfere with the effect of *Rab39b* deficiency on brain growth. The fact that not all patients carrying loss-of-function *RAB39B* mutations have macrocephaly (Supplementary Table 1) also suggest that loss of *Rab39b* does not necessarily lead to macrocephaly in mice; and other factors may coordinate with *Rab39b* deficiency for this abnormality development. Alternatively, this discrepancy may be caused by the different mouse ages studied. It was reported that mouse cortical structural growth had a near exponential growth rate initially but tapered off at postnatal days 15–20; and subsequently the growth rate kept at a steady state (Baloch et al., 2009). Therefore, it is possible that loss of *Rab39b* boosts brain growth dramatically during an early brain developmental stage and thus results in significant brain size difference between WT and *Rab39b* KO mice when the brain growth rate reaches the peak (e.g., at postnatal day 20). While after this stage, both WT and *Rab39b* KO mouse brain growth rates slow down and the effect of *Rab39b* deficiency on NPC over-proliferation also decreases as active NPC numbers drop during brain development, narrowing the gap between WT and *Rab39b* KO mouse brain sizes in adults.

Autophagy is a conserved mechanism for degrading unnecessary or abnormal cytoplasmic entities to maintain cellular homeostasis in response to stress. Autophagy dysregulation may result in neurodevelopmental as well as neurodegenerative diseases (Lee et al., 2013; Frake et al., 2015; Beltran et al., 2019; Lachance et al., 2019). Herein, we found that RAB39B deficiency resulted in elevated LC3B-II levels by impairing autophagic flux at basal level; and this is consistent with the elevated PI3K-AKT-mTOR signaling upon loss of *Rab39b* since this mTOR signaling inhibits autophagy (Dibble and Cantley, 2015). Coincidently, the C9ORF72/WDR41/SMCR8/ATG101 complex acts as a GDP/GTP exchange factor for RAB39B and deficiency in components of this complex can also alter autophagy, implying that such alterations may be mediated by RAB39B (Sellier et al., 2016; Yang et al., 2016; Corbier and Sellier, 2017; Tang et al., 2020). Moreover, we found that treatment with the mTOR inhibitor rapamycin comparably stimulated autophagy in both control and RAB39B-deficient cells, suggesting that RAB39B deficiency impairs basal autophagic flux but fails to affect cellular response to autophagy simulation. Interestingly, we noticed that levels of p62, another commonly used autophagy marker were not changed in *Rab39b* KO mice compared to WT controls (Figure 4A and Supplementary Figure 6). Since p62 regulates diverse processes such as apoptosis and necroptosis and interacts with several signaling molecules that affect p62 transcriptional synthesis (Sahani et al., 2014; Sanchez-Martin et al., 2019), p62 may be dispensable for canonical autophagy in *Rab39b* KO mice; and this deserves further scrutiny.

Upregulation of autophagy has been found to reverse disease-like phenotypes in animal models of FCD, PD, AD, etc. (Lee et al., 2013; Frake et al., 2015; Zhao S. et al., 2019). Herein, we also found that rapamycin treatment improved novel object recognition memory and rescued LTP deficits in *Rab39b* KO mice, suggesting that rapamycin may also alleviate symptoms in XLID patients caused by *RAB39B* mutations.

AMPA receptors are the most common excitatory glutamate receptors in the brain, which are tetramers composed of four subunit types (GluA1–GluA4) and can be directly activated upon glutamate binding. AMPA receptors primarily mediate rapid electrophysiological responses to glutamate. NMDA receptors mainly exist in the postsynaptic membrane and are also tetramers composed of two GluN1 subunits together with either two GluN2 subunits or one GluN2 and one GluN3 subunits. NMDA receptor activation by glutamate requires either glycine or D-serine as a co-agonist, and is relatively slow and prolonged compared to AMPA receptor activation (Scannevin and Huganir, 2000; Hettinger et al., 2018; Hanada, 2020). A previous study found that RAB39B could mediate trafficking of the AMPA receptor subunit GluA2 and downregulation of RAB39B skewed AMPA receptor composition toward non-GluA2-containing calcium-permeable forms, affecting mouse hippocampal neuron synaptic activity (Mignogna et al., 2015). In the PSD of *C9orf72* KO mice whose RAB39B GDP/GTP exchange factor is impaired, levels of RAB39B were decreased, whereas levels of the AMPA receptor subunit GluA1 were increased (Xiao et al., 2019), implying that RAB39B may affect GluA1 trafficking. However, we found that in *Rab39b* KO mice, the PSD distribution of NMDA receptors rather than AMPA receptors were significantly decreased. The discrepancy between our and others’ results may be attributed to the different cell and mouse lines used. Nevertheless, electrophysiological studies revealed decreased NMDA/AMPA ratios and unaltered AMPA-eEPSC amplitudes in our *Rab39b* KO mice compared to WT controls, strengthening a functional deficiency of NMDA receptors rather than AMPA receptors in these mice. Moreover, rapamycin treatment partially rescued memory and LTP deficits without obviously affecting NDMA receptor distribution, therefore, the contribution of defective autophagy and NMDA receptors to synaptic functions and learning and memory in *Rab39b* KO mice may be executed through different mechanisms.

**FIGURE 6:**
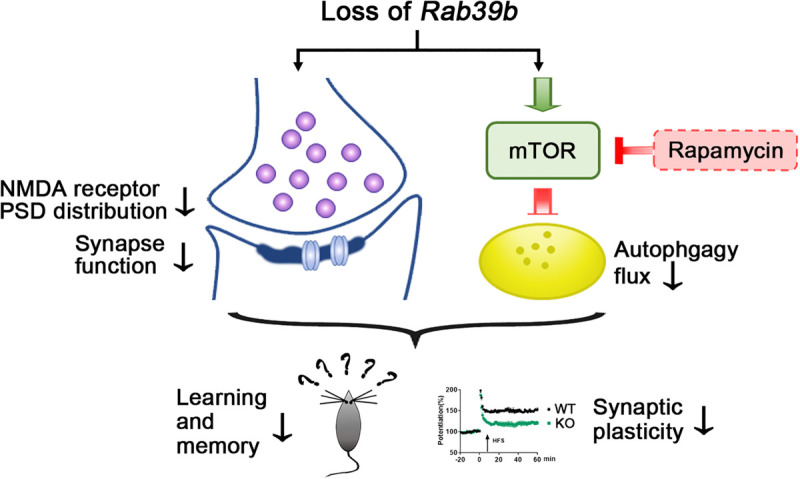
Scheme of RAB39B deficiency induced synaptic and learning and memory impairment. Deletion of *Rab39b* results in defective synaptic structure and function and PSD distribution of NMDA receptors, as well as increased mTOR signaling and compromised autophagy flux. Rapamycin treatment stimulates autophagy through inhibiting mTOR and partially rescues impaired memory and synaptic plasticity in *Rab39b* KO mice.

In summary, our study demonstrates that the neuron-specific protein RAB39B plays a crucial role in regulating learning and memory. Deletion of *Rab39b* leads to synaptic dysfunction and autophagy disturbance. Rapamycin treatment can partially rescue impaired memory and synaptic plasticity in *Rab39b* KO mice (Figure 6). These findings not only demonstrate the importance of RAB39B in learning and memory through modulating both autophagy and synapse formation, but also suggest that targeting autophagy holds potential for intervention of learning and memory deficits in patients carrying *RAB39B* mutations. In the current study we only carried out research in young mice. Since *RAB39B* mutations are also associated with the neurodegenerative disease PD, the age-dependent changes in *Rab39b* KO mice and whether these changes can be reversed by modulating autophagy and/or glutamate receptors warrant further scrutiny.

## Data Availability Statement

The raw data supporting the conclusions of this article will be made available by the authors, without undue reservation, to any qualified researcher.

## Ethics Statement

The animal study was reviewed and approved by the Animal Ethics Committee of Xiamen University.

## Author Contributions

MN and YZ designed the research. MN, ZW, XL, XF, YW, ML, TY, PY, JM, and YZ conducted the molecular, cellular, and animal experiments. NZ, YG, ZC, and TW performed the electrophysiological experiments. YG, ZW, and QM made intellectual contributions. MN, HX, and YZ wrote the manuscript. YZ supervised the project. All authors reviewed the manuscript.

## Conflict of Interest

The authors declare that the research was conducted in the absence of any commercial or financial relationships that could be construed as a potential conflict of interest.
