# Supplementary material for: RAB39B Deficiency Impairs Learning and Memory Partially Through Compromising Autophagy
Source: Front Cell Dev Biol. 2020 Dec 8;8:598622. doi: 10.3389/fcell.2020.598622 (PMC7753041; doi:10.3389/fcell.2020.598622)
Supplement: Supplementary file 1 [file Table_1.DOCX]

**Supplementary Table 1** Loss-of-function *RAB39B* mutations associated with diseases.

| **Mutations** | **Mutation types** | **Clinical phenotypes** | **References** |
| --- | --- | --- | --- |
| c.21C>A (p.T7X) | Truncation | Mild to severe ID, macrocephaly, some with ASD | Giannandrea et al., 2010 |
| c.215+1G>A (5’ splice site) | Splicing is affected to disrupt protein biosynthesis | Moderate to severe ID, some with ASD and seizures | Giannandrea et al., 2010 |
| Loss of *RAB39B* locus | Loss of *RAB39B* expression | Early onset PD, ID, macrocephaly | Wilson et al., 2014 |
| c.503C>A (p.T168K) | Mutant is destabilized | Early onset PD, ID, some with seizures and macrocephaly | Wilson et al., 2014 |
| c.574G>A (p.G192R) | Mutant is mislocalized | Early onset PD, some with ID | Mata et al., 2015 |
| c.428C>G (p.A143G) | Missense mutation predicted to be deleterious | PD | Mata et al., 2015 |
| c.624_626delGAG (p.R209del) | Deletion mutation predicted to be deleterious | PD | Mata et al., 2015 |
| c.557G>A (p.W186X) | Truncation | Early onset PD, mild ID | Lesage et al., 2016 |
| c.536dupA (p.E179fsX48) | Frameshift and truncation | Early onset PD, mild ID | Shi et al., 2016 |
| c.432delA (p.A144fsX3) | Frameshift and truncation | Progressive parkinsonism, ID | Guldner et al., 2016 |
| c.559G>T (p.E187X) | Truncation | ID, macrocephaly, ASD | Woodbury-Smith et al., 2017 |
| c.137dupT (p.S47fsX44) | Frameshift and truncation | Early onset PD, ID | Ciammola et al., 2017 |
| c.371delA (p.K124fsX10) | Frameshift and truncation | Early onset PD, ID, ASD | Ciammola et al., 2017 |
| c.436_447del (p.G146_Y149del) | In-frame deletion | ID, tremor | Santoro et al., 2020 |
