# Supplementary material for: RAB39B Deficiency Impairs Learning and Memory Partially Through Compromising Autophagy
Source: Front Cell Dev Biol. 2020 Dec 8;8:598622. doi: 10.3389/fcell.2020.598622 (PMC7753041; doi:10.3389/fcell.2020.598622)
Supplement: Supplementary file 3 [file Data_Sheet_1.pdf]

RAB39B deficiency impairs learning and memory partially through compromising autophagy

Supplementary Materials

Supplementary MATERIALS AND METHODS

Rab39b KO mouse generation and genotyping

Mouse *Rab39b* gene has two exons, with the protein coding sequence (CDS) spanning both exons (Figure S2a). A TALEN-mediated strategy to target *Rab39b* exon 1 was utilized to generate *Rab39b* KO mice, with the service provided by Cyagen Biosciences Inc. The left (targeting 5’-TTCACCGAGGGCCGCTTT -3’) and right (targeting 5’-TCTACCCCCACGGTGGGA-3’) TALEN arms were designed and constructed (Figure S2b). Constructs were transcribed *in vitro*, and TALEN mRNAs were injected into fertilized C57BL/6J mouse eggs to generate *Rab39b* KO animals. For mouse genotyping, genomic DNA was extracted from mouse tails by dissolving in 0.02 M NaOH at 98°C for 30 min and then neutralizing with 1 M Tris-HCl (pH 8.0). PCR primers used for genotyping were: 5’-CGATCTCCACCAAACGGGAG-3’ and 5’-GTGGGCAAGTCCTGCCTGAT-3’. An 103

bp fragment and an 101 bp fragment were PCR-amplified from wild type and *Rab39b* KO alleles, respectively, and resolved using a 15% Native-PAGE.

Western blot

Mouse tissue samples were lysed in RIPA lysis buffer [150 mM NaCl, 25 mM Tris–HCl, pH 8.0, 0.5% (wt/vol) sodium deoxycholate, 0.05% (wt/vol) sodium dodecyl sulfate, and 1% (vol/vol) Nonidet P-40] supplemented with protease and phosphatase inhibitor cocktails for 40 min. Cells were lysed in TNEN lysis buffer [50 mM Tris–HCl, 150 mM NaCl, pH 8.0, 2 mM EDTA, and 1% (vol/vol) Nonidet P-40] supplemented with protease and phosphatase inhibitor cocktails for 30 min. Equal amounts of protein lysates were resolved using SDS-polyacrylamide gel electrophoresis, transferred to polyvinylidene difluoride membranes (Merck Millipore), probed with primary antibodies and then secondary antibodies as indicated, and detected by chemiluminescence.

Mouse behavioral tests

Nine cohorts of mice were subjected to behavioral experiments with the following order: open field tests, novel object recognition tests, high elevated plus maze tests, T-maze tests, three-chamber social interaction tests, Morris water maze tests, rotarod tests, and hanging tests.

Open field tests were used to detect locomotor activity and anxiety behavior of mice.

The open field box is a rectangular box (40 cm (L) x 40 cm (W) x 40 cm (H)). The mouse exploratory activity was recorded for 10 min by Smart 3.0 video tracking system (Panlab). Total distance travelled in the arena and time spent in the center were measured for comparison.

High elevated plus maze tests were used to detect mouse anxiety. The maze is composed of two open arms and two closed arms forming a "+" shape (15 cm (L) x 6 cm (W)). Mice were placed in the center of the maze facing to an open arm. The exploratory activity was recorded for 5 min. Mouse exploring time in open arms was recorded by Smart 3.0 camera system for comparison.

T-maze tests were used to measure spontaneous alternation behaviors that indicate short-term working memory. The maze has three equally spaced arms forming a "T" shape (30cm (L) × 6cm (W) × 15cm (H)). Mice were placed in the center of the maze and their exploratory activity was recorded for 5 min. Sequence of arm entries and total number of arms entered were recorded by Smart 3.0.

Novel object recognition tests contain habituation, training and testing phases. Mice were first allowed to habituate to an open field box (40 cm (L) x 40 cm (W) x 40 cm (H)) for 10 min. In the training phase, mice were allowed to explore the open field box with two identical objects (A and B) for 10 min. After 24 h, object B was replaced with a novel object

(C) and mice were tested for their exploring times to objects A and C for 10 min. Mouse exploration time to each object was counted for comparison.

Morris water maze is a circular tank (120 cm in diameter) filled with tap water at a temperature of 22±2°C and contains an underwater platform. Walls surrounding the tank are taped with different shapes of pictures that serve as visual reference cues. The experiment was divided into two parts. In the training part, mice were placed into water to let them swim, and the time mice used to find and climb onto the platform was recorded. The maximum time mice allowed to swim were 60 s. If mice could not find the platform within 60 s, they were guided to the platform and allowed to stay on the platform for 5 s. Mice were trained for five days and on each day, mice were trained for four times by placing into the water from four different positions (N, S, W, and E). In the testing part, the underwater platform was removed on the 6th day and mice were placed into water to let them search the underwater platform. The percentage time mice spent in each of the four quadrants was recorded by Smart 3.0 for comparison.

Three-chamber social interaction tests were used to detect the social interaction behavior of mice. The social box is a rectangle box (60 cm (L) x 42 cm (W) x 22 cm (H)) and divided into three equal chambers (left, middle, and right) along its long side by two transparent plastic plates. There is a door on the plate to allow mice move between chambers.

There is a small cage in each of the two side chambers. Firstly, mice were placed in the middle chamber with both cages empty to let mice explore the whole box freely for 5 min. After a stranger mouse was put into the cage in the left chamber, tested mice were allowed to continue exploring the whole box for another 10 min. Finally, another stranger mouse was put into the cage in the right chamber, and tested mice were allowed to continue exploring the whole box for another 10 min. Mouse exploring time to each cage at different stages were recorded by Smart 3.0 for comparison.

Rotarod tests were used to measure motor and balance ability of mice. Mice were trained on the rotarod at 4 rpm until their latency time to fall was longer than 30 sec before testing. In the testing phase, mice were placed on the rotarod starting at 4 rpm and the rotarod was accelerated at a frequency of 5 rpm/min. The maximum time for mice to stay on the rod was 120 s. Mice were given three tests a day for three consecutive days. The latency time to fall was recorded by PenLab.

Hanging tests were used to detect the griping power of mice. Mice were made to grab and hang on a square wire mesh placed at a height of 50 cm above the ground, and the maximum time for mice to hang upside down was 180 s. The latency time to fall was recorded for comparison.

Electrophysiology

Ice-cold solution (64 mM NaCl, 2.5 mM KCl, 10 mM glucose, 1.25 mM NaH_2PO_4, 10 mM $MgSO_4$, 26 mM $NaHCO_3$, 120 mM sucrose, and 0.5 mM $CaCl_2$) was prepared and frozen in -80 °C 1 h before experiments. Mice were anesthetized and mouse brains were quickly dissected by scissors and cut into 400 μm thick coronal slices by vibrating microtome (Leica VT1200S) in ice-cold solution aerated with 95% O_2/5% CO_2. Slices were placed into artificial cerebrospinal fluid (aCSF) containing 126 mM NaCl, 3.5 mM KCl, 1.25 mM NaH_2PO_4, 1.3 mM $MgSO_4$, 2.5 mM $CaCl_2$, 26 mM $NaHCO_3$, and 10 mM glucose. Slices were recovered for 1 h at 34°C and then at room temperature for an additional 2-8 h in aCSF. All solutions were saturated with 95% O_2/5% CO_2. Evoked-excitatory postsynaptic current (eEPSC) amplitudes were recorded in the CA1 region when different stimulus intensities (0.2, 0.4, 0.6, 0.8, 1.0, 1.2, 1.4, 1.6, 1.8, and 2.0 mA) were applied in the CA3 region. Paired-pulse ratio was measured by different inter-pulse intervals (10, 20, 50, 100, and 200 ms) in the CA3 region. LTP in the CA1 was recorded after the CA3 area was induced by two trains of 100-HZ stimuli (with 30 s interval).

Evoked EPSCs (eEPSCs) were recorded by stimulating the schaffer collateral pathway with a two-concentric bipolar stimulating electrode (FHC, Inc) positioned ~ 250 μm from CA1 pyramidal neurons. 5 mM lidocaine N-ethylchloride (QX-314) was added in the pipette. AMPA and NMDA receptor-mediated eEPSCs were recorded at a holding potential of -70 mV and +40 mV, respectively. 100 μM picrotoxin was added in the extracellular solution.

The resistance of pipettes was 5-8 MΩ. Data were filtered at 0.5 kHz and sampled at 10 kHz, and acquired with a patch-clamp amplifier (Multiclamp 700B, Molecular Devices) and analyzed using pClamp 10.6 software (Molecular Devices). NMDA/AMPA receptor response ratios were calculated for comparison. Alternatively, AMPA receptor-mediated eEPSCs was recorded at a holding potential of -70 mV and stimulated with increased intensities at 10, 30, 60, 90, 120, 150, 180, 210, 240, 270 and 300 μA. 100μM picrotoxin and 50 μM D-AP5 was added in the extracellular solution.

Electron microscope analysis

After anaesthetized, mice were perfused with saline followed by fixative solution (4%(wt/vol) paraformaldehyde and 2.5%(vol/vol) glutaraldehyde in 0.1 M phosphate buffer, pH 7.4). of Mouse cortical V1/V2 and hippocampal CA1 regions were dissected and stored with a fixative solution supplemented with 4%(wt/vol) paraformaldehyde and 1%(vol/vol) glutaraldehyde in 0.1 M phosphate buffer (pH 7.2) for 48 h. Tissues were washed in phosphate-buffered saline, followed by incubating with 2%(wt/vol) osmium tetroxide for 1 h and dehydrating in a graded series of ethanol solutions. Samples were embedded in Epon resin. After polymerization, ultra-thin sections were cut, stained with 2.5%(wt/vol) uranyl acetate and 1%(wt/vol) lead citrate, and deposited on electron microscope grids for examination under a JEOL 1011 (Tokyo, Japan) transmission electron microscope.

Preparation of synaptosomal and PSD fractions

Mouse tissues were dissected and homogenized in cold sucrose buffer (0.32 M sucrose and 25 mM HEPES, pH 7.4). Homogenates were centrifuged at 1,400 g for 10 min to separate the supernatant (S1; total). The S1 fraction was centrifuged at 10,000 g for 12 min to separate the supernatant (S2; light membrane and cytosolic fraction) and the precipitation (P2; crude synaptosomal fraction). The P2 fraction was washed with sucrose buffer and re-suspended in cold HBS buffer (25 mM HEPES, pH 7.4, and 150 mM NaCl) to get the synaptosomal (Syn) fraction. The synaptosomal fraction was re-suspended in sucrose buffer with 1%(vol/vol) Triton X-100 at 4°C for 40 min, followed by 40,000 g centrifugation for 30 min to obtain the precipitation (PSD fraction).

Dopamine ELISA Assay

Dopamine concentrations in mouse midbrain were quantified by ELISA using Mouse DA ELISA Kits (Meimian, Cat#MM-0626M1), following the manufacturer's instructions.

Supplementary FIGURE LEGENDS

Supplementary FIGURE 1 Mouse RAB39B expression pattern. **(A)** RAB39B protein levels in various tissues of 2-month-old wild type C57BL/6J mice were detected by western blot. **(B)** RAB39B protein levels in different brain tissues of 2-month-old wild type C57BL/6J mice were detected by western blot, quantified by densitometry, and normalized to those of β-actin for comparison. Relative RAB39B levels in cortex were set to one arbitrary units (A.U.). Data represent mean ± SEM, $n = 4$, ns: not significant, Mann-Whitney test. **(C)** RAB39B in mouse primary neurons, microglia, and astrocytes were detected by western blot, quantified by densitometry, and normalized to those of β-actin for comparison. Relative RAB39B levels in neurons were set to one A.U. Data represent mean ± SEM, $n = 4$, $^{*}p<0.05$, Mann-Whitney test. NeuN, Iba-1, and GFAP were detected to evaluate the purity of neurons, microglia, and astrocytes, respectively. **(D)** RAB39B, GluN1, and synapsin 1 (SYN1) proteins in brain lysates of C57BL/6J wild type mice at different postnatal days were detected by western blot. RAB39B levels were quantified by densitometry and normalized to those of β-actin for comparison. Relative RAB39B levels at P0 were set to one A.U. Data represent mean ± SEM, $n = 4$, ns: not significant, $^{*}p < 0.05$, Mann-Whitney test.

Supplementary FIGURE 2 Alignment of human and mouse RAB39B protein sequences. A different amino acid site is highlighted in green.

Supplementary FIGURE 3 Generation of *Rab39b* knockout (KO) mice. **(A)** Scheme of the mouse *Rab39b* gene. *Rab39b* has two exons and its protein coding sequence (CDS) spans both exons (225-866, nomenclature according to National Center for Biotechnology Information Reference Sequence: NM_175122). **(B)** Two TALEN spacers (TALEN-L and TALEN-R) designed to integrate mutations into *Rab39b* exon 1 are shown (in green). The two nucleotides "GT" (CDS sites 106-107) deleted in the generated mouse line are indicated in red. **(C)** Sequencing the "GT" deletion sites in wild type ($Rab39b^{+/+}$ or $Rab39b^{+/Y}$), heterozygous female ($Rab39b^{+/-}$), and homozygous female ($Rab39b^{-/-}$) or hemizygous ($Rab39b^{-/Y}$) KO mice. **(D)** Genotyping of wild type (WT), heterozygous female, homozygous female, and hemizygous male *Rab39b* KO mice. Amplified PCR products spanning the targeted mutation sites were subjected to SDS-PAGE analysis to resolve WT and mutant (Mut) fragments. **(E)** RNAs of WT and *Rab39b* KO mouse brain were extracted and reverse transcribed. *Rab39b* mRNA levels were detected by qRT-PCR and normalized to those of β-actin for comparison (WT controls were set to one arbitrary units, A.U.). Data represent mean

± SEM, $n = 4$ for each group, $^{*}p < 0.05$, Mann-Whitney test. **(F)** The fluorescence intensity of NeuN staining in cortical and hippocampal regions in Figure 1f were measured by ImageJ and compared to respective WT controls (set to one A.U.). Data represent mean ± SEM, n= 5 for each group, ns: not significant, Mann-Whitney test.

Supplementary FIGURE 4 Loss of *Rab39b* has no effect on mouse locomotor activity and muscle strength. **(A)** WT and *Rab39b* KO mice were analyzed for their mean moving speed, total travel distance, and duration in the center in open field tests. **(B)** WT and *Rab39b* KO mice were analyzed for their total travel distance and mean swimming speed in water maze tests. **(C)** WT and *Rab39b* KO mice were studied for their hanging time and impulses in four-limb hanging tests. Data represent mean ± SEM, $n = 13$ for each group, ns: not significant, Mann-Whitney test.

Supplementary FIGURE 5 Loss of *Rab39b* has no effect on synaptic vesicle numbers and total levels of synapse-related proteins. **(A)** Synaptic vesicle numbers in Figure 3G were counted for comparison. Data represent mean ± SEM, $n = 15$ neurons from 3 mice per group, ns: not significant, Mann-Whitney test. **(B)** Equal protein quantities of cortex or hippocampus lysates derived from WT and *Rab39b* KO mice were subjected to western blot to study the

proteins indicated.

Supplementary FIGURE 6 RAB39B deficiency impairs autophagy. Equal protein quantities of cortex and midbrain lysates derived from WT and *Rab39b* KO mice were subjected to western blot for the proteins indicated. Levels of p62, LC3B-II and S6 phosphorylated at S240/244 (p-S6) were quantified by densitometry and normalized to those of β-actin or S6, respectively, and compared to WT controls (set to one arbitrary units, A.U.). Data represent mean ± SEM, $n = 4$ for each group, $^{*}p < 0.05$, Mann-Whitney test.

Supplementary FIGURE 7 Rapamycin treatment does not affect locomotor activity, short-term working memory, or reduced anxiety in *Rab39b* KO mice. **(A-C)** *Rab39b* KO mice treated with DMSO vehicle or rapamycin were compared for their total travel distance and duration in the center in open field tests **(A)**, their spontaneous alternation in T maze tests **(B)**, and their time staying in the open arm and total travel distance in high elevated plus maze tests **(C)**. Data represent mean ± SEM, $n = 9$ in open field and T maze tests for each group, $n = 7$ in high elevated plus maze tests for each group, ns: not significant, Mann-Whitney test.

Supplementary FIGURE 8 RAB39B deficiency affects tyrosine hydrolysase (TH) and dopamine levels. **(A)** Equal protein quantities of midbrain lysates derived from WT and *Rab39b* KO mice were subjected to western blot for the proteins indicated. Levels of TH were quantified by densitometry and normalized to those of β-actin, respectively, and compared to WT controls (set to one arbitrary units, A.U.). Data represent mean ± SEM, n = 4 for each group, $^{*}p < 0.05$, Mann-Whitney test. **(B)** Dopamine levels in WT and *Rab39b* KO mouse midbrain were determined by ELISA for comparison. $n = 4$, $^{*}p < 0.05$, Mann-Whitney test.
