# Supplementary figures and images for: RAB39B Deficiency Impairs Learning and Memory Partially Through Compromising Autophagy
Source: Front Cell Dev Biol. 2020 Dec 8;8:598622. doi: 10.3389/fcell.2020.598622 (PMC7753041; doi:10.3389/fcell.2020.598622)

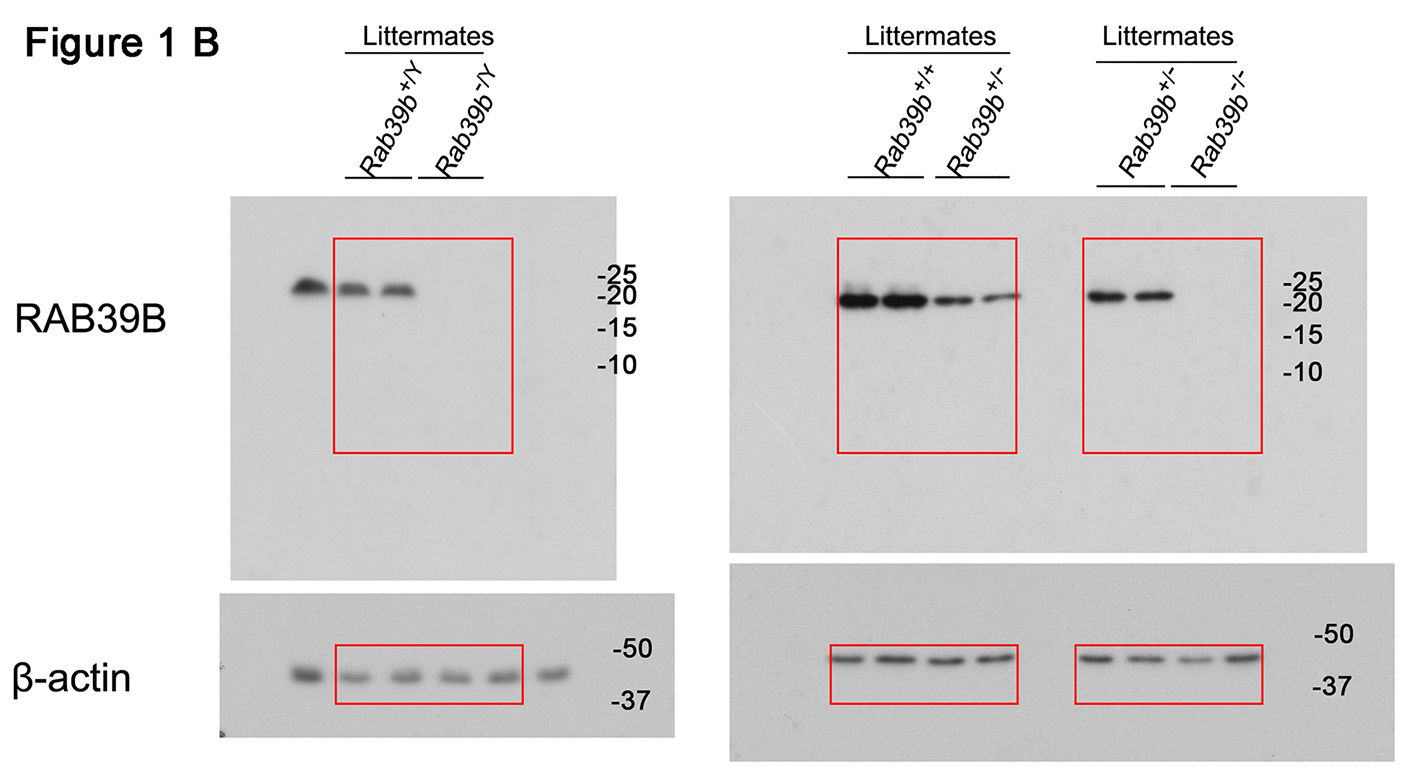

Supplement: Supplementary file 4 [file Data_Sheet_2.ZIP › raw data/Figure 1-new.tif]

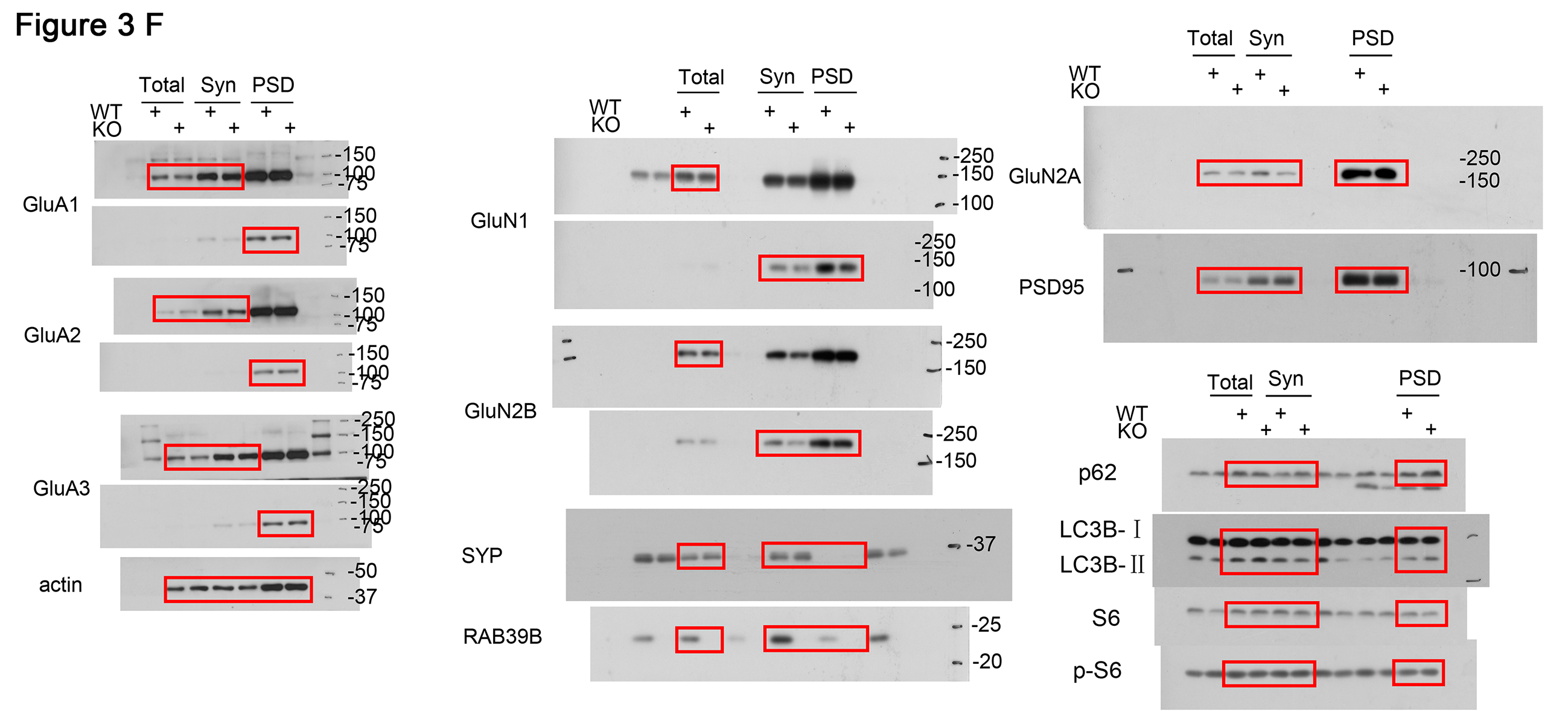

Supplement: Supplementary file 4 [file Data_Sheet_2.ZIP › raw data/Figure 3-new.tif]

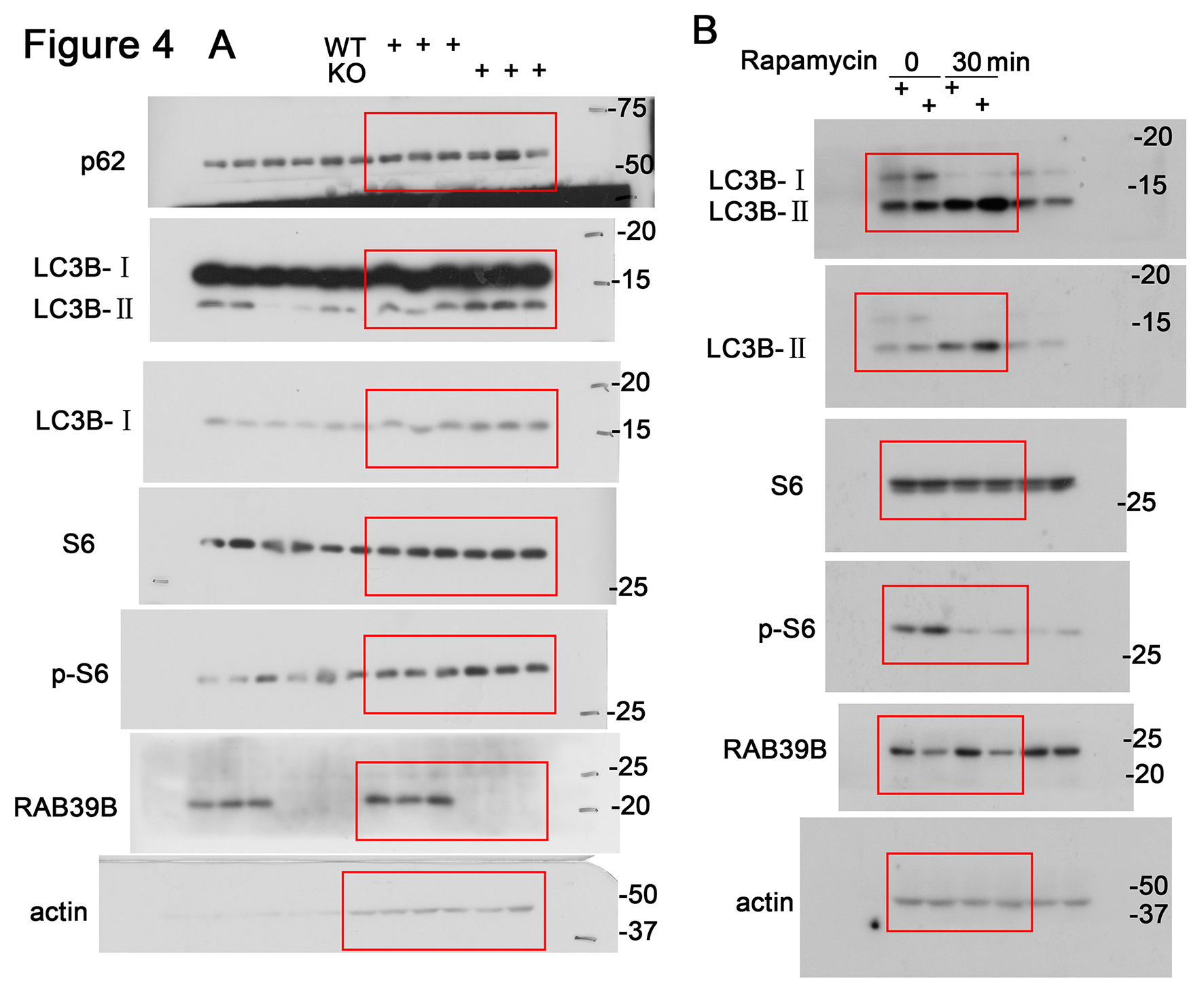

Supplement: Supplementary file 4 [file Data_Sheet_2.ZIP › raw data/Figure 4-new.tif]

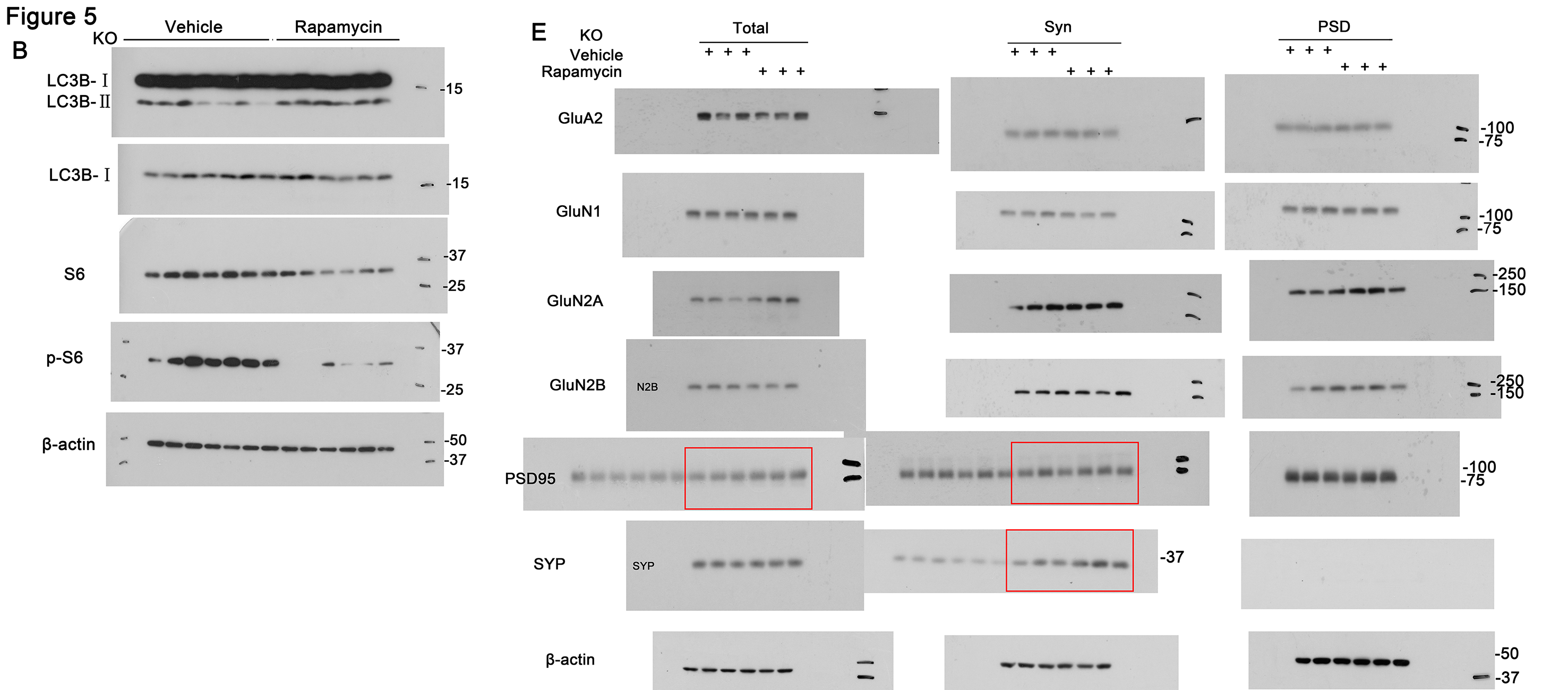

Supplement: Supplementary file 4 [file Data_Sheet_2.ZIP › raw data/Figure 5-new.tif]

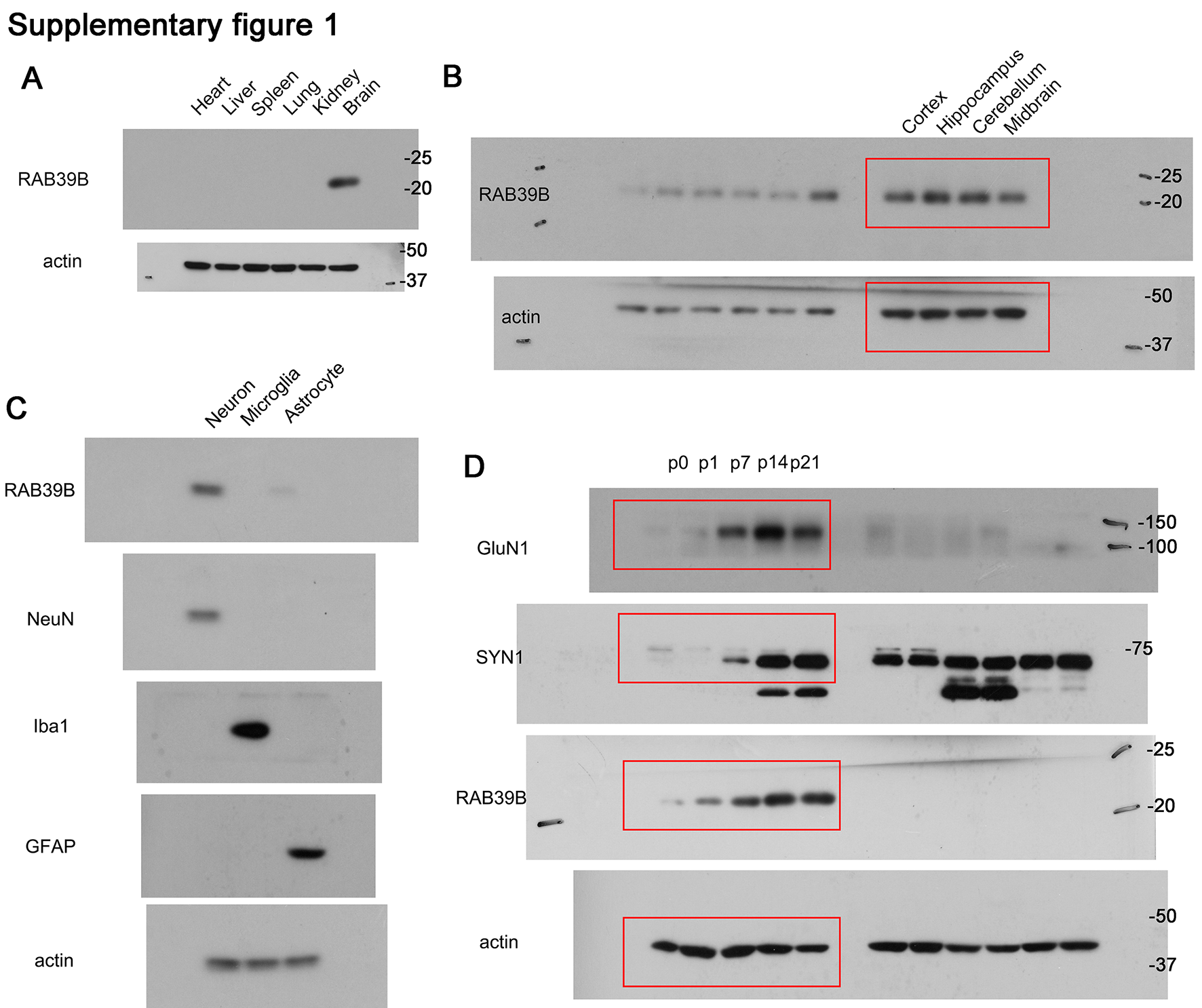

Supplement: Supplementary file 4 [file Data_Sheet_2.ZIP › raw data/Supplementary figure 1-new.tif]

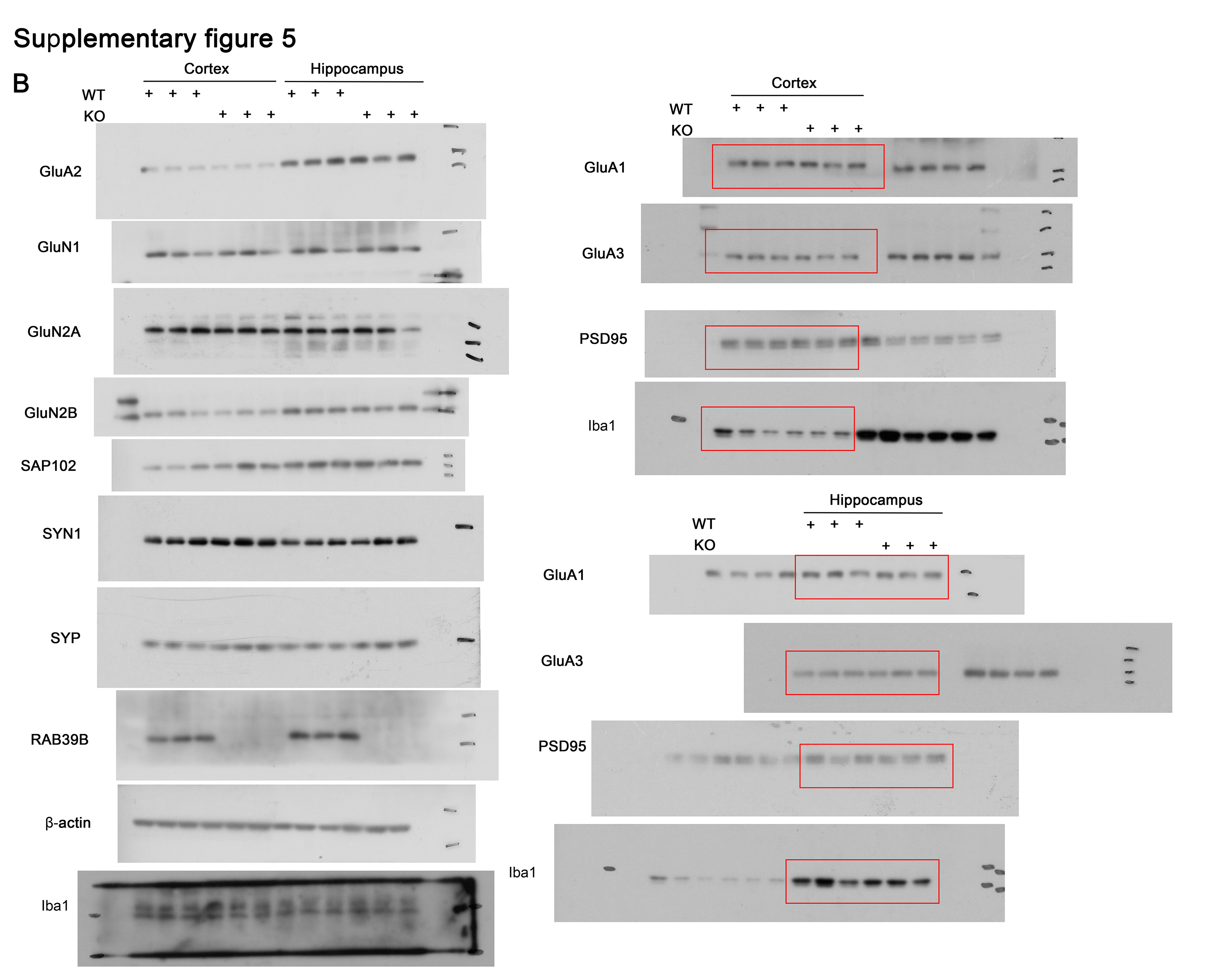

Supplement: Supplementary file 4 [file Data_Sheet_2.ZIP › raw data/Supplementary figure 5-new.tif]

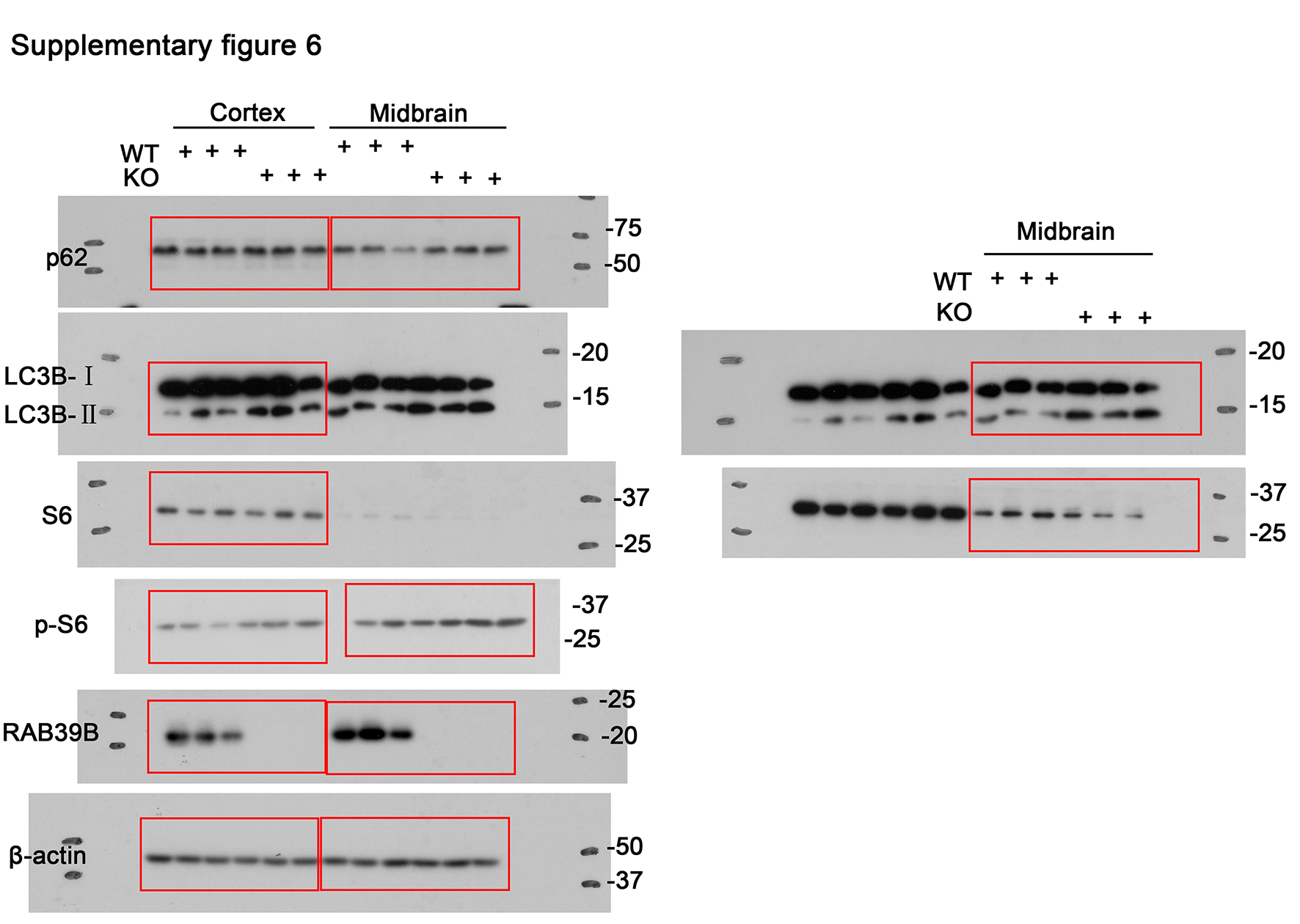

Supplement: Supplementary file 4 [file Data_Sheet_2.ZIP › raw data/Supplementary figure 6-new.tif]

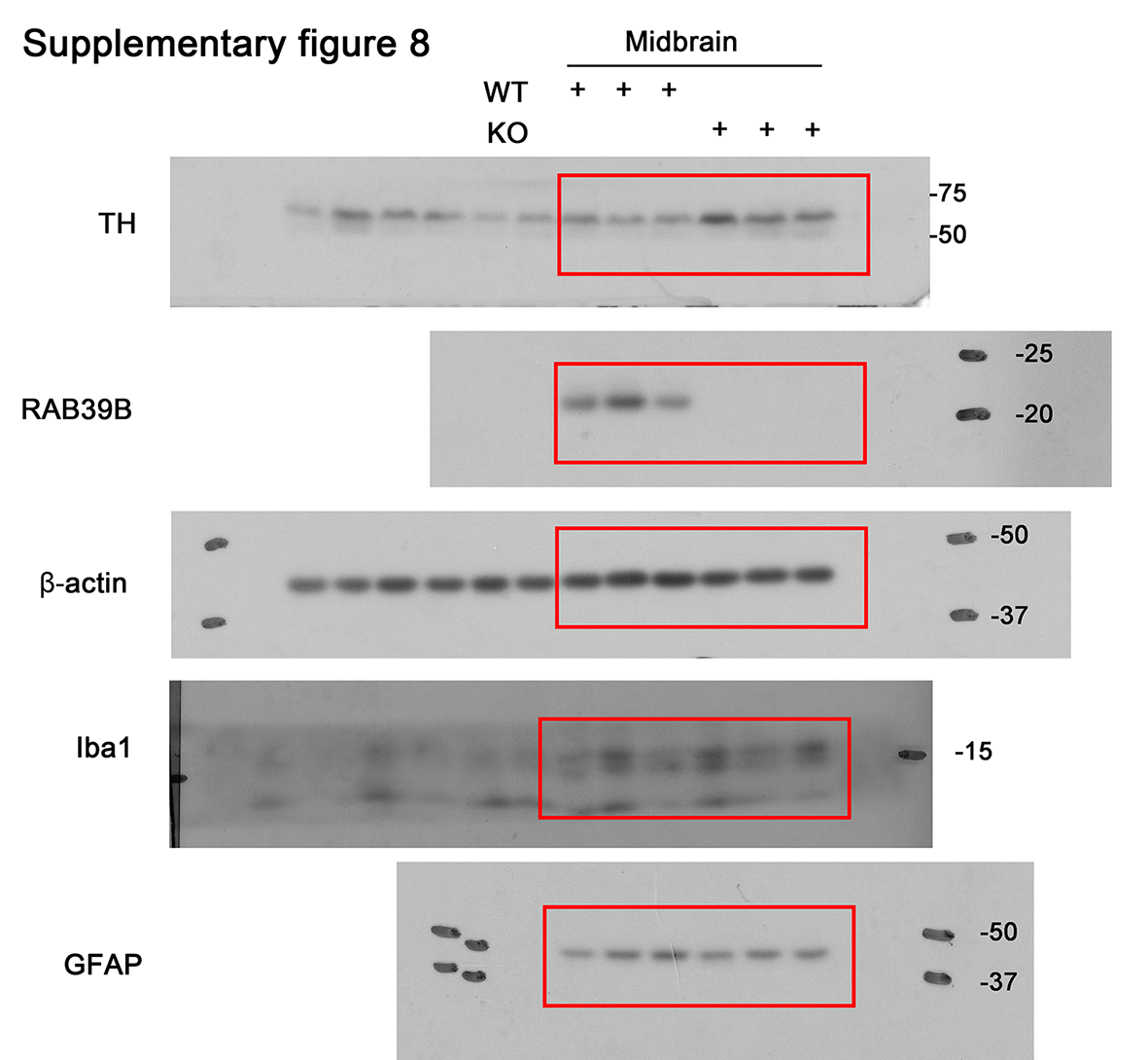

Supplement: Supplementary file 4 [file Data_Sheet_2.ZIP › raw data/Supplementary figure 8-new.tif]

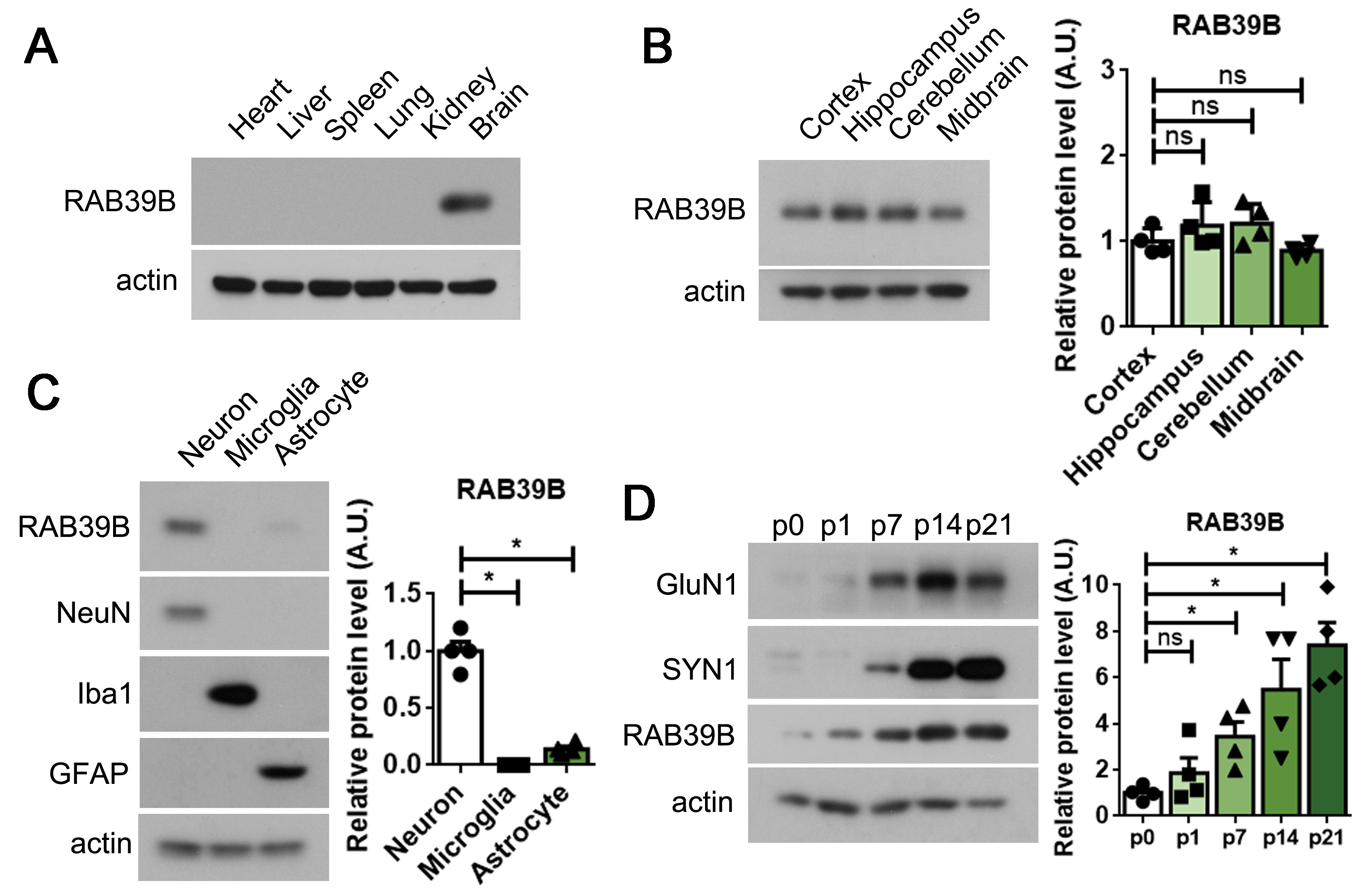

Supplement: Supplementary file 6 [file Image_1.TIF]

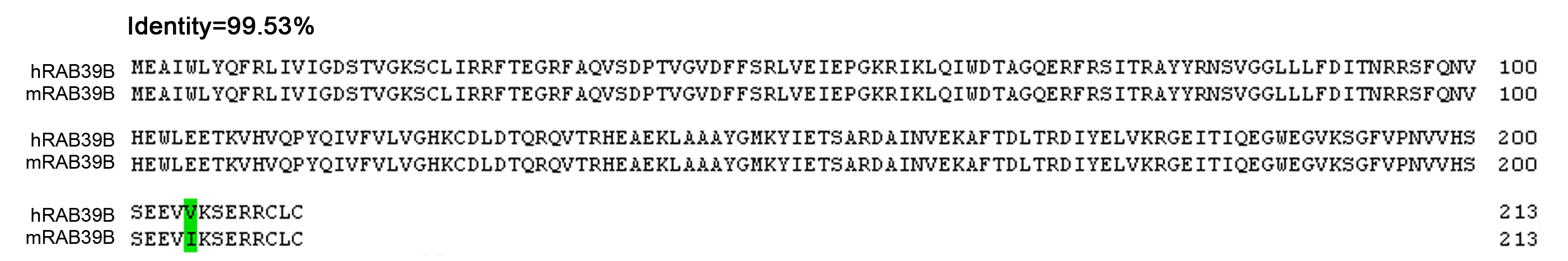

Supplement: Supplementary file 7 [file Image_2.TIF]

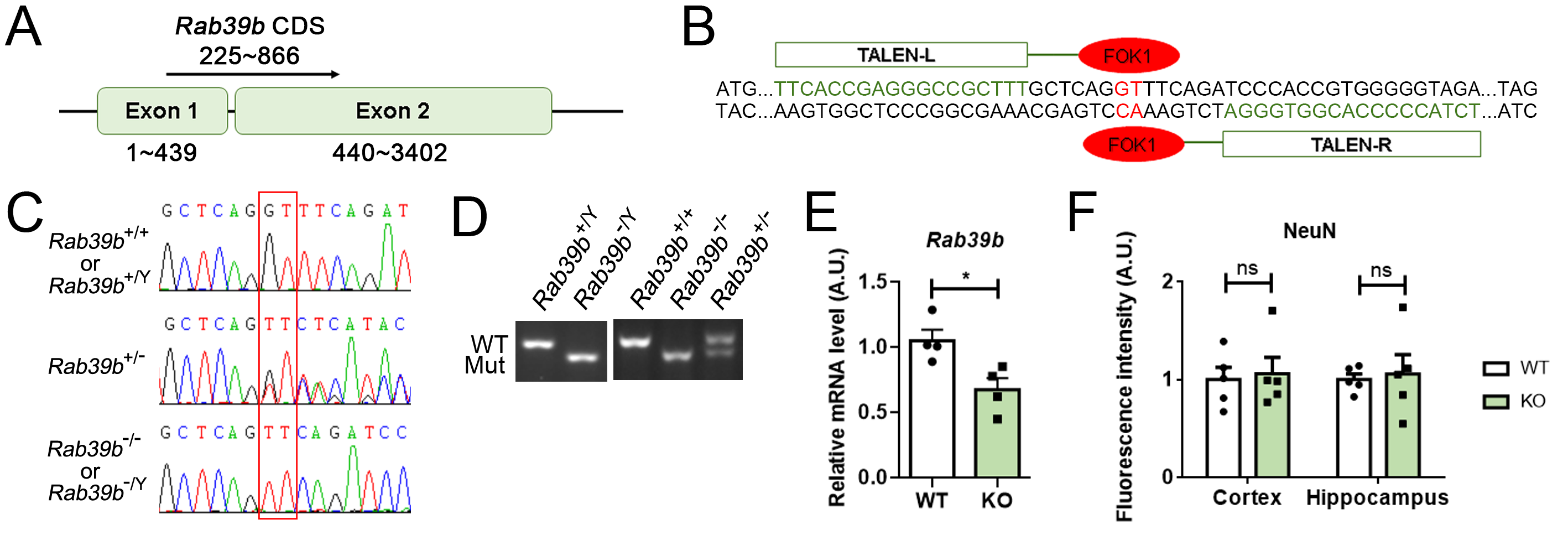

Supplement: Supplementary file 8 [file Image_3.TIF]

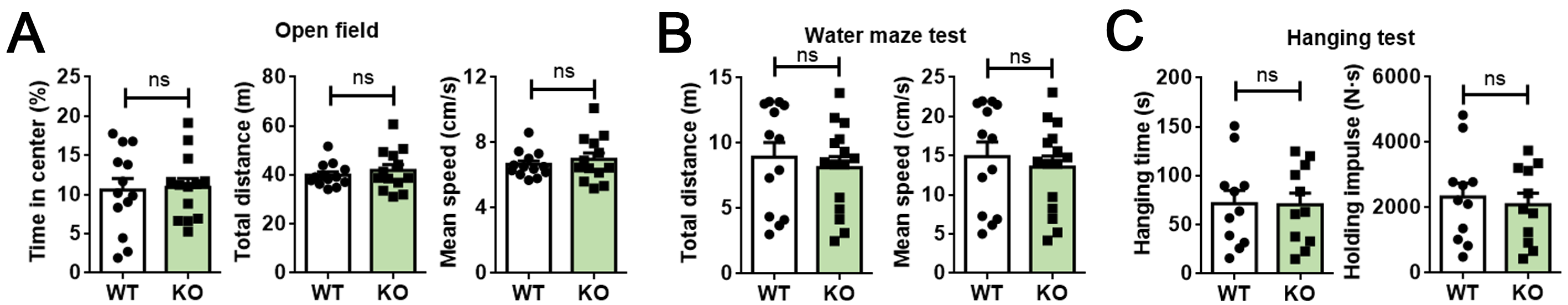

Supplement: Supplementary file 9 [file Image_4.TIF]

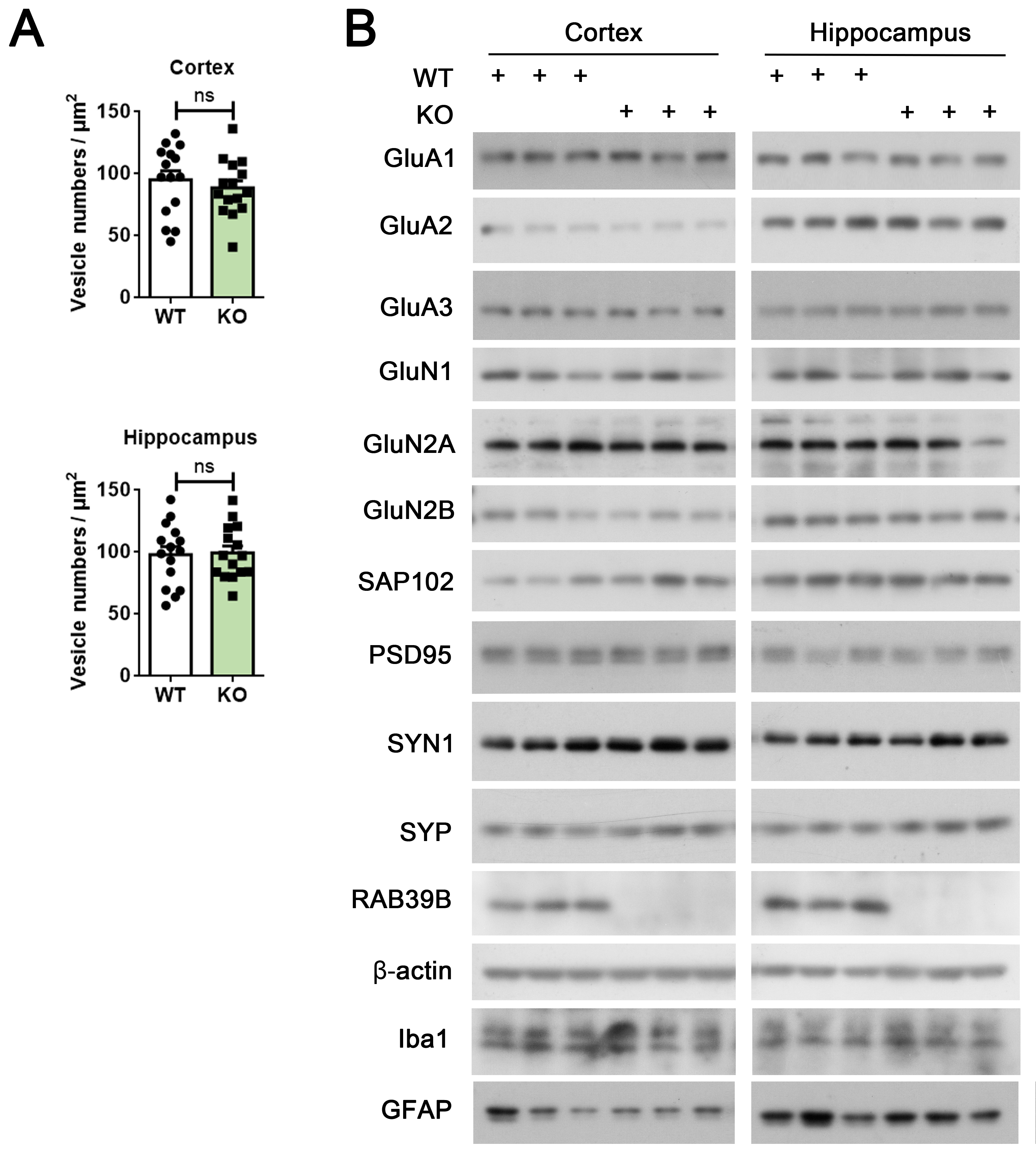

Supplement: Supplementary file 10 [file Image_5.TIF]

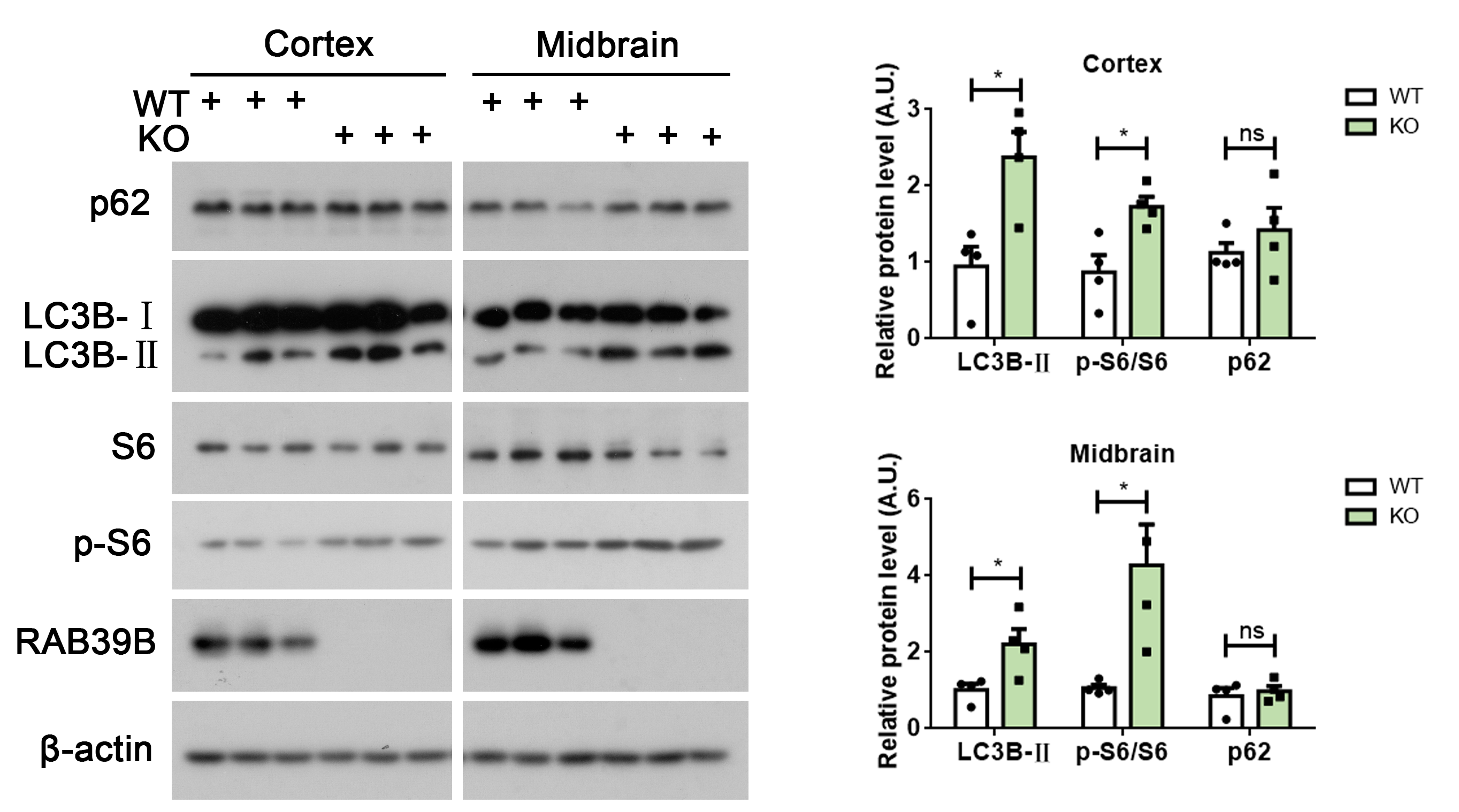

Supplement: Supplementary file 11 [file Image_6.TIF]

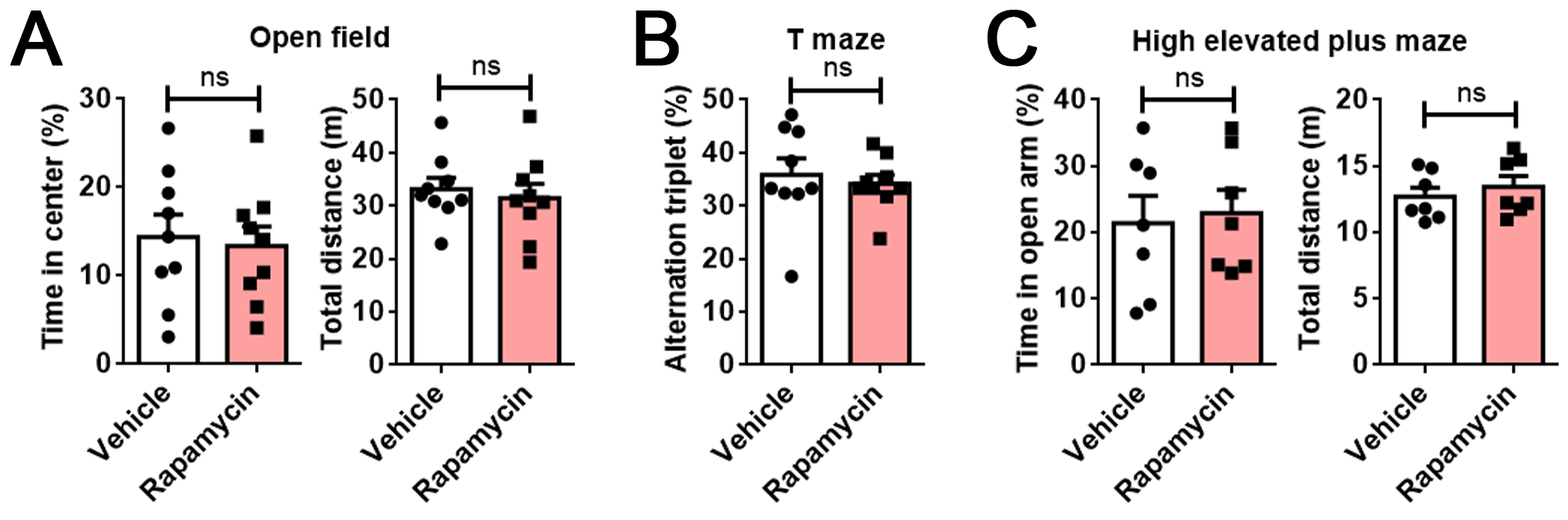

Supplement: Supplementary file 12 [file Image_7.TIF]

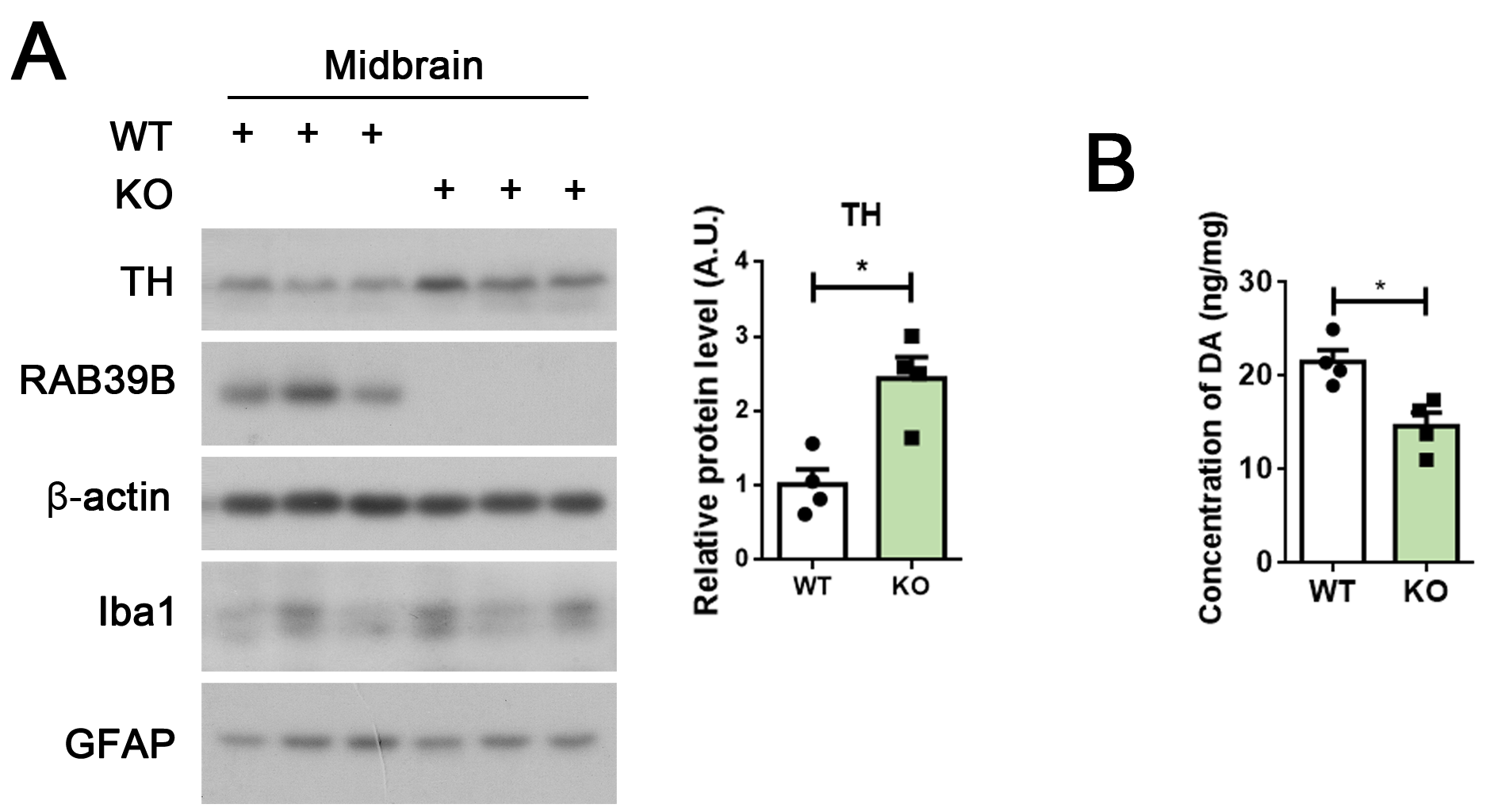

Supplement: Supplementary file 13 [file Image_8.TIF]
