# Supplementary material for: RAB39B Deficiency Impairs Learning and Memory Partially Through Compromising Autophagy
Source: Front Cell Dev Biol. 2020 Dec 8;8:598622. doi: 10.3389/fcell.2020.598622 (PMC7753041; doi:10.3389/fcell.2020.598622)
Supplement: Supplementary file 5 [file Data_Sheet_3.PDF]

RAB39B deficiency impairs learning and memory partially through compromising autophagy

Mengxi Niu[1,2], Naizhen Zheng[2], Zijie Wang[3], Yue Gao[2], Xianghua Luo[2], Zhichai Chen[2], Xing Fu[2], Yanyan Wang[1], Ting Wang[2], Manqing Liu[1], Tingting Yao[2], Peijie Yao[2], Jian Meng[2], Yunqiang Zhou[2], Yunlong Ge[3], Zhanxiang Wang[4], Qilin Ma[1], Huaxi Xu[2], Yun-wu Zhang[1,2*]

[1]Department of Neurology, The First Affiliated Hospital of Xiamen University, Xiamen, Fujian 361003, China

[2]Fujian Provincial Key Laboratory of Neurodegenerative Disease and Aging Research, Institute of Neuroscience, School of Medicine, Xiamen University, Xiamen, Fujian 361102, China

[3]Department of Neurosurgery, Xiang'an Hospital of Xiamen University, Xiamen, Fujian 361102, China

[4]Department of Neurosurgery, The First Affiliated Hospital of Xiamen University, Xiamen, Fujian 361003, China

[*]**Correspondence:** Yun-wu Zhang (E-mail: yunzhang@xmu.edu.cn)

ABSTRACT

RAB39B is located on the X chromosome and encodes the RAB39B protein that belongs to the RAB family. Mutations in *RAB39B* are known to be associated with X-linked intellectual disability (XLID), Parkinson's disease, and autism. However, the patho/physiological functions of RAB39B remain largely unknown. In the present study, we established *Rab39b* knockout (KO) mice, which exhibited overall normal birth rate and morphologies as wild type mice. However, *Rab39b* deficiency led to reduced anxiety and impaired learning and memory in 2-month-old mice. Deletion of *Rab39b* resulted in impairments of synaptic structures and functions, with reductions in NMDA receptors in the postsynaptic density (PSD). RAB39B deficiency also compromised autophagic flux at basal level, which could be overridden by rapamycin-induced autophagy activation. Further, treatment with rapamycin partially rescued impaired memory and synaptic plasticity in *Rab39b* KO mice, without affecting the PSD distribution of NMDA receptors. Together, these results suggest that RAB39B plays an important role in regulating both autophagy and synapse formation, and that targeting autophagy may have potential for treating XLID caused by *RAB39B* loss-of-function mutations.

Keywords: Autophagy, Learning and memory, NMDA receptors, RAB39B, Rapamycin

INTRODUCTION

Intellectual disability (ID) refers to a group of neurodevelopmental disorders characterized by severe defects in both cognition (with an IQ score of less than 70) and adaptive behavior, which start before the age of 18 years (Vissers et al., 2016; Iwase et al., 2017). Although environmental factors such as maternal drug abuse during pregnancy and birth complications can cause ID, increasing evidence indicates that genetics also plays a significant role in the pathogenesis of ID (Vissers et al., 2016; Iwase et al., 2017). It is estimated that genomic/genetic variants contribute to about 25%-50% of ID incidence. X-linked ID (XLID) resulting from genomic/genetic variants on X chromosome is one of the most common ID types and has a prevalence of 10%-15% in the whole ID population (Peng et al., 2017). Because of the hemizygosity of X chromosomes in males, XLID occurs much more in males than females. So far genomic/genetic variants in more than one hundred genes on X chromosome have been associated with XLID (Peng et al., 2017). However, detailed molecular pathways underlying ID pathogenesis driven by each of these genes remain largely unknown; and elucidation of underlying molecular mechanisms shall provide new insights into disease therapeutic strategies.

The *RAB39B* gene is located on X chromosome (Xq28) and encodes the RAB39B protein that belongs to the Rab protein family (Cheng et al., 2002). Similar to other small GTPases,

the activity of Rab proteins is regulated by GTP-bound active and GDP-bound inactive states. As an evolutionarily conserved vesicle transporter regulator group, Rab proteins localize to different membrane structures to regulate vesicle trafficking (Corbeel and Freson, 2008). Recent studies have found that loss-of-function *RAB39B* mutations are associated with various diseases including XLID, autistic spectrum disorder (ASD), and Parkinson's disease (PD) (Supplementary Table 1) (Giannandrea et al., 2010; Mata et al., 2015; Guldner et al., 2016; Lesage et al., 2016; Shi et al., 2016; Ciammola et al., 2017; Woodbury-Smith et al., 2017; Santoro et al., 2020). However, the physiological functions of RAB39B as well as its pathological roles in disease pathogenesis remain largely elusive.

Several reports suggested that RAB39B may regulate protein trafficking and the PI3K-AKT-mTOR pathway. On one hand, one study found that RAB39B could interact with PICK1 to control trafficking of the AMPA receptor subunit GluA2 from the endoplasmic reticulum to the Golgi and then to cell surface; downregulation of RAB39B skewed AMPA receptor composition towards non-GluA2-containing calcium-permeable forms and hence altered synaptic activity of mouse hippocampal neurons (Mignogna et al., 2015). Consistently, downregulation of RAB39B in neurons altered the number and morphology of neurite growth cones and reduced presynaptic buttons, suggesting a role of RAB39B in synapse formation and maintenance (Giannandrea et al., 2010). On the other hand, RAB39B was found to interact with PI3K components and loss of RAB39B could promote PI3K-AKT-mTOR activity,

resulting in altered cortical neurogenesis, macrocephaly and ASD-like behaviors in mice (Zhang et al., 2020). Nevertheless, whether loss of RAB39B lead to various disease phenotypes through the same mechanism has yet to be determined.

MATERIALS AND METHODS

Animals

Rab39b KO mice were generated as described in Supplementary Materials and Methods. C57BL/6J wild type (WT) mice were provided by Xiamen University Laboratory Animal Center. All animal procedures were approved by the Animal Ethics Committee of Xiamen University and were conducted in accordance with the National Institutes of Health Guidelines for the Care and Use of Laboratory Animals. The mice used were all 2 month old, except otherwise indicated specifically.

Antibodies, reagents, and western blot

Antibodies against RAB39B (Cat# D-12162-1-AP), GluA2 (Cat# 11994-1-AP), GluN2A (Cat# 19953-1-AP), GluN2B (Cat# 19954-1-AP), and tyrosine hydroxylase (TH, Cat# 66334-1-IG) were from Proteintech. Antibodies against GFAP (Cat# 3670s), β-actin (Cat# 8457s), GluN1 (Cat# 5704s), PSD95 (Cat# 3450s), LC3B (Cat# 3868s), S6 (Cat# 2217s), and

phosphorylated S6 (Ser240/244) (Cat# 5364s) were from Cell Signaling Technology. Antibodies against GluA1 (Cat# 04-855), GluA3 (Cat# MAB5416), and SYN1 (Cat# AB1543) were from Millipore. Antibodies against NeuN (Cat# ab177487), Iba1 (Cat# 016-20001), and synaptophysin (SYP) (Cat# S5768) were from Abcam, Wako, and Sigma-Aldrich, respectively. Goat anti-rabbit (Cat# 31460) or anti-mouse (Cat# 31430) IgG (HCL) secondary antibodies conjugated with horseradish peroxidase were from Thermo Fisher Scientific.

The mTOR inhibitor rapamycin (Cat# HY-10219) was from MedChemExpress. DMSO (Cat# 20688) was from Thermo Fisher Scientific. 4′, 6-diamidino-2-phenylindole (DAPI) (Cat# D9542) was from Sigma-Aldrich. Complete Protease Inhibitor and PhosSTOP Cocktails were from Roche.

Different protein levels in mouse tissues and cells were determined by western blot. Detailed procedures are presented in Supplementary Materials and Methods. Protein band intensity was quantified by densitometry using the Image J software (National Institutes of Health).

Cell culture

Mouse neuroblastoma N2a cells were maintained in high glucose DMEM (Hyclone) supplemented with 10 %(vol/vol) fetal bovine serum (FBS, Gibco), 100 units/ml penicillin

(Gibco), and 100 μg/ml streptomycin (Gibco), and incubated at 37°C in humidified air with 5% CO_2.

RNA interference

An siRNA sequence (sense: 5'-UCAUUCUUCAGAAGAGGUUTT-3'; antisense: 5'-AACCUCUUCUGAAGAAUGATT-3') targeting mouse *Rab39b* was designed and synthesized by GenePharma. A scrambled siRNA sequence (sense: 5'-UUCUCCGAACGUGUCACGUTT-3'; antisense: 5'- ACGUGACACGUUCGGAGAATT-3') was used as a negative control. These siRNAs were labelled with the fluorescent dye Cy5 and were transfected into N2a cells using Lipofectamine 3000 Reagent (Invitrogen), following the manufacturer's protocol.

DNA construct and transfection

The RFP-GFP-LC3B vector was from Biovector Science Lab, Inc. The construct was transfected into N2a cells by Turbofect transfection reagent (Thermo Fisher Scientific), following the manufacturer's protocol.

Quantitative real time-PCR (qRT-PCR)

Total RNAs were extracted using the TRIzol Reagent (Invitrogen), and reverse-transcribed using the ReverTra Ace qPCR RT Kit (Toyobo), following the manufacturers' instructions.

Equal amounts of cDNA from each sample were subjected to qRT-PCR. Primers used were as follows:

For *Rab39b*:

forward primer: 5’-CTGGGATACAGCGGGTCAAG-3’;

reverse primer: 5’-GAAGGACCTGCGGTTGGTAA-3’;

For *β-actin*:

forward primer: 5’-AGCCATGTACGTAGCCATCCA-3’;

reverse primer: 5’-TCTCCGGAGTCCATCACAATG-3’.

Immunofluorescence

Mouse brain sections were treated with citrate buffer (pH 7.0) for 10 min for antigen retrieval, permeabilized and blocked in PBS containing 0.5%(vol/vol) Triton X-100 and 10%(vol/vol) normal goat serum at room temperature for 1 h. After washing with PBS for 3 times, samples were incubated with primary antibodies in blocking buffer overnight at 4°C, and then with fluorescence-conjugated secondary antibodies in blocking buffer at room temperature for 1 h. After PBS washing, samples were stained with DAPI for 10 min. Images were acquired using a confocal fluorescence microscope (Nikon).

Cells transfected with *Rab39b* siRNA and RFP-GFP-LC3B were fixed in 4%(wt/vol) paraformaldehyde for 15 min. After washing with PBS, cells were stained with DAPI for 10

min and visualized under a confocal fluorescence microscope (Nikon). The fluorescence intensity was quantified by densitometry using Image J.

Mouse behavioral tests

Detailed procedures for behavioral experiments are presented in Supplementary Materials and Methods.

Electrophysiology

Electrophysiological recordings were performed as previously described (Wen et al., 2010; Wen et al., 2011; Zeng et al., 2019). More details are provided in Supplementary Materials and Methods.

Golgi staining

Freshly dissected mouse brains were subjected to Golgi staining using the FD Rapid Golgi Stain system (FD Neuro Technologies), following the manufacturer's instructions. Brains were sliced with a vibratome (Leica) at a thickness of 100 μm. After dehydration, Golgi-stained neurons and spines were visualized under a confocal microscope (Nikon). Apical distal spine density of neurons in cortical V1/V2 and hippocampal CA1 regions were quantified by densitometry using Image J.

Electron microscope analysis

Synapse structures were assayed using a transmission electron microscope (Ung et al., 2018; Zhao et al., 2019a). More details are provided in Supplementary Materials and Methods.

Preparation of synaptosomal and PSD fractions

PSD fractions from mouse hippocampus were dissected as previously described (Wang et al., 2013). More details are provided in Supplementary Materials and Methods.

Rapamycin treatment

Rapamycin treatment procedure was based on a previously reported study (Zhou et al., 2009). Briefly, rapamycin was first dissolved in DMSO (100 mg/ml) and then diluted with 0.9%(wt/vol) saline to 1 mg/ml. DMSO vehicle were dissolved in 0.9% saline to a concentration of 1%(vol/vol). 2-month-old *Rab39b* KO mice were intraperitoneally injected with rapamycin at 7.5 mg/kg or DMSO vehicle at 7.5 ml/kg per day for 7 consecutive days. Starting from the 8th day, mice were subjected to electrophysiological analysis or behavioral tests, during which processes mice were injected with rapamycin continually. On the 14th day, mice were sacrificed for subsequent biochemical analysis.

Statistics

Data represent mean ± standard error of means (SEM). Statistical analysis was performed using Graphpad Prism 6 or SPSS 13.0 softwares. Detailed statistical data and methods for each comparison are indicated in the text and/or in Supplementary Table 2.

RESULTS

Generation and characterization of *Rab39b* knockout (KO) mice

RAB39B was previously reported as a neural-specific protein (Giannandrea et al., 2010). Here we also confirmed that RAB39B was specifically expressed in the brain but not other tissues detected in 2-month-old C57BL/6J mice (Supplementary Figure 1A). The expression level of RAB39B was comparable in different brain regions including cortex, hippocampus, cerebellum, and midbrain (Supplementary Figure 1B). Moreover, we found that mouse RAB39B was predominantly expressed in primary neurons, with minimal detection in primary astrocytes and no detection in primary microglia (Supplementary Figure 1C). At early postnatal stages, the expression of mouse RAB39B in the brain was gradually increased and showed a pattern correlated well to those of neuronal proteins GluN1 and synapsin 1 (Supplementary Figure 1D).

RAB39B is highly conserved between mouse and human, with only one amino acid

difference (Supplementary Figure 2), suggesting their functional conservation and that outcome from mouse study may predict authentic functions of human RAB39B. Therefore, to study the physiological function of RAB39B *in vivo*, we used the transcription activator-like effector nucleases (TALEN) technique to generate *Rab39b* KO mice in a C57BL/6J background and obtained a mouse line that had a two nucleotide "GT" deletion within the *Rab39b* protein coding sequence (CDS sites 106-107) (Supplementary Figures 3A-D). Such a deletion resulted in a frame shift and early truncation of RAB39B (Figure 1A). RAB39B was undetectable in either hemizygous *Rab39b* KO male mice or homozygous *Rab39b* KO female mice (Figure 1B), indicating the loss of wild type RAB39B in this mouse line. Mutant *Rab39b* mRNA levels was also decreased compared to WT *Rab39b* mRNA levels (Supplementary Figure 3E). In addition, RAB39B protein levels were relatively low in heterozygous *Rab39b* KO female mice compared to WT littermate female mice (Figure 1B).

Both *Rab39b* KO male and female mice were viable and fertile. When heterozygous *Rab39b* KO female mice were crossed with hemizygous *Rab39b* KO male mice, the genotypes (chi-squared test, p = 0.83) as well as the sex ratio (chi-squared test, p = 0.73) of the offspring followed a Mendelian frequency (Figure 1C).

Because *RAB39B* is an X-linked gene with a recessive inheritance and X-linked intellectual disability is more common in males, we focused our study on male mice and unless

specific mentioning, mice used throughout the study refer to males only. The overall body and brain morphologies of *Rab39b* KO mice were indistinguishable from those of littermate controls at 2 months of age (Figure 1D). The body weight but not the brain weight of *Rab39b* KO mice were lighter than those of littermate controls at 2 months of age, resulting in an increased brain/body weight ratio in *Rab39b* KO mice (Figure 1E). Moreover, we found no obvious neuronal abundance differences in cortical and hippocampal regions between *Rab39b* KO and WT mice at 2 months of age (Figure 1F and Supplementary Figure 3F). These results suggest that RAB39B is not essential for normal development in mice.

Altered behavioral phenotypes in *Rab39b* KO mice

In open field tests, *Rab39b* KO mice showed no differences in moving speed, the time spent in the center, and total travel distance compared to WT littermates (Supplementary Figure 4A). However, in high elevated plus maze tests, *Rab39b* KO mice spent more time in the open arms than WT littermates (Figure 2A), implying that loss of *Rab39b* reduced mouse anxiety.

Since loss of function mutations in *RAB39B* cause ID, autism, and PD symptoms in humans, we next studied whether loss of *Rab39b* affects mouse behaviors resembling these diseases. In novel object recognition tests, both *Rab39b* KO and WT mice spent similar time exploring the two identical objects during the training (Figure 2B, left panel). However, although WT mice spent more time exploring the novel object than the familiar object, *Rab39b*

KO mice explored the novel and the familiar objects with similar time (Figure 2B, right panel), suggesting that loss of *Rab39b* impaired recognition memory. Moreover, *Rab39b* KO mice showed reduced spontaneous alternations compared to WT mice in T-maze tests (Figure 2C), indicating that loss of *Rab39b* also impaired short-term working memory. During Morris water maze tests, WT and *Rab39b* KO mice had similar total travel distance and swimming speed (Supplementary Figure 4B). However, despite exhibiting little difference from WT controls in their escape latency to the hidden platform during the training (Figure 2D, left panel), *Rab39b* KO mice spent significantly less time in the target quadrant than WT controls during the testing (Figure 2D, right panel), suggesting impaired spatial memory in *Rab39b* KO mice.

In three-chamber social interaction tests, we found that both *Rab39* KO mice and WT mice spent significantly more time approaching the cage with a strange mouse (Stranger 1) than the empty cage (Figure 2E, left panel). However, when another strange mouse (Stranger 2) was placed into the empty cage, WT mice preferred to explore Stranger 2, whereas *Rab39b* KO mice showed no such a preference (Figure 2E, right panel). These results suggest that *Rab39b* deficiency mainly impairs social novelty recognition rather than sociability.

In rotarod tests, mice were given three tests per day for three consecutive days. WT and *Rab39b* KO mice stayed on the rod for similar time periods during the first four tests. But then WT mice had increased latency for staying on the rod in following tests, whereas *Rab39b* KO

mice failed to improve the latency (Figure 2F). In four-limb hanging tests which assess rodents' muscle strength, *Rab39b* KO mice showed similar ability in hanging time and hanging impulse compared to WT mice (Supplementary Figure 4C). These results suggest that loss of *Rab39b* compromises motor skill learning but does not affect motor ability at a young age.

Rab39b **KO mice exhibit defects in synaptic function and structure and PSD composition**

Since impaired learning and memory is associated with altered synaptic plasticity (Acquarone et al., 2019; Zhao et al., 2019a), we carried out electrophysiological study on synaptic functions. When evoked excitatory postsynaptic currents (eEPSCs) in the hippocampal CA1 stratum radiatum were recorded by stimulating the Schaffer collateral (SC)/commissural pathway at various intensities, a marked reduction in eEPSC slopes in *Rab39b* KO mice was detected compared to WT mice (Figure 3A). Since the ratio of paired-pulse facilitation was not significantly different between *Rab39b* KO and WT mice (Figure 3B), the reduced eEPSCs in *Rab39b* KO mice are probably caused by post-synaptic defects. LTP at the SC-CA1 region was also dramatically reduced in *Rab39b* KO mice compared to WT mice (Figure 3C).

AMPA receptors and NMDA receptors play crucial roles in synaptic function and memory formation (Scannevin and Huganir, 2000; Hettinger et al., 2018; Hanada, 2020). To characterize potential differences of the two receptors in *Rab39b* KO mice, we measured NMDA/AMPA receptor response ratios and evoked AMPA excitatory postsynaptic currents

(AMPA-eEPSCs) in hippocampal CA1 neurons of WT and *Rab39b* KO mice. We found that NMDA/AMPA receptor response ratios were significantly decreased in *Rab39b* KO neurons compared to WT controls (Figure 3D), whereas AMPA-eEPSC amplitudes were not significantly altered upon loss of *Rab39b* (Figure 3E). These results suggest that NMDA receptor function rather than AMPA receptor function is impaired in *Rab39b* KO mice.

We next evaluated and found significant spine density reductions in both cortical and hippocampal neurons of *Rab39b* KO mice when compared to their littermate controls (Figure 3F). We also performed an ultra-structure analysis of post-synaptic density (PSD) by transmission electron microscopy and found that both PSD length and width in the cortex and hippocampal CA1 regions of *Rab39b* KO mice were significantly reduced compared to WT controls (Figure 3G), whereas synaptic vesicle numbers in both regions were not different (Supplementary Figure 5A).

We next studied and found that the total amounts of AMPA and NMDA receptor subunits showed no differences between *Rab39b* KO and WT mice (Figure 3H and Supplementary Figure 5B). However, the PSD fraction distribution of NMDA receptors including GluN1, GluN2A, and GluN2B were marked reduced in *Rab39b* KO mice (Figure 3F), whereas the PSD distribution of AMPA receptors including GluA1, GluA2, and GluA3 remained unchanged (Figure 3H). These findings are consistent with the specific NMDA receptor

function impairment in *Rab39b* KO mice as revealed by electrophysiological studies (Figures 3D,E). Together, these results indicate that RAB39B plays an important role in maintaining synaptic plasticity and structure and PSD composition.

Loss of *Rab39b* impairs autophagy

One recent study found that RAB39B regulates the PI3K-AKT-mTOR signaling (Zhang et al., 2020). Here we also found that phosphorylated S6 levels were increased in the hippocampus, cortex, and midbrain of *Rab39b* KO mice compared to WT controls (Figure 4A and Supplementary Figure 6), corroborating an upregulation of the PI3K-AKT-mTOR signaling upon loss of RAB39B. Since the PI3K-AKT-mTOR pathway inhibits autophagy, we further studied whether RAB39B regulates autophagy. Levels of LC3B-II, an autophagy marker were increased in *Rab39b* KO mice (Figure 4A and Supplementary Figure 6). Consistently, downregulation of RAB39B in mouse N2a cells resulted in increased levels of phosphorylated S6 and LC3B-II (Figures 4B, C). However, rapamycin treatment induced LC3B-II increase and phosphorylated S6 decrease in both control and RAB39B-downregulated cells, with comparable levels (Figure 4C). Although an increase of LC3B-II levels usually suggests autophagy activation, LC3B-II level increase may also be caused by a deficiency in the autophagosome-lysosome fusion that blocks autophagic flux and thus LC3B-II degradation in the autophagolysosome. Therefore, we downregulated RAB39B in N2a cells and then

transfected them with an RFP-GFP-LC3B construct. The advantage of using this RFP-GFP-LC3B construct is that the GFP signal but not the RFP signal is quenched in the acidic and degradative autophagolysosome, so that a change of the GFP/RFP ratio indicates a change of the autophagolysosome formation (Kimura et al., 2007). Herein, we found that the GFP/RFP signal ratio was increased in RAB39B-downregulated cells compared to controls (Figure 4D), suggesting that RAB39B deficiency caused a reduction in autophagolysosome formation and thus a decreased autophagic flux; and this is consistent with the elevated PI3K-AKT-mTOR signaling. However, when these cells were treated with rapamycin, the ratio of GFP/RFP signal in both control and RAB39B-downregulated cells were similarly reduced (Figure 4D). Together, these results indicate that RAB39B deficiency impairs autophagic flux at basal level but does not affect cellular response to autophagy stimulation.

Rapamycin treatment partially rescues memory and LTP defects in *Rab39b* KO mice

To investigate whether promoting autophagy could rescue impaired memory and defective synaptic plasticity in *Rab39b* KO mice, we treated mice with rapamycin or DMSO vehicle by intraperitoneal injection (Figure 5A). We found that phosphorylated S6 levels were decreased and LC3B-II levels were increased in the hippocampal tissues of *Rab39b* KO mice treated with rapamycin when compared to those treated with vehicle (Figure 5B), indicating that autophagy was induced in mouse hippocampus by rapamycin treatment. Importantly, *Rab39b*

KO mice treated with rapamycin showed significantly improved memory in novel object recognition tests when compared to controls (Figure 5C), while rapamycin treatment had no effect on affecting their locomotor activity in open field tests (Supplementary Figure 7A) or reversing the compromised short-term working memory in T maze tests (Supplementary Figure 7B), and the decreased anxiety in high elevated plus maze tests (Supplementary Figure 7C). Moreover, rapamycin treatment significantly improved impaired LTP in *Rab39b* KO mice (Figure 5D). However, the PSD distribution of NMDA receptor subunits (GluN1, GluN2A, and GluN2B) was not affected by rapamycin treatment (Figure 5E).

DISCUSSION

Gao *et al.* recently generated *Rab39b* KO mice (in C57BL/6J background) using the CRISPR/Cas9 technique. By comparing *Rab39b* KO and WT mice, they found that the expression of mouse RAB39B was high in the cortex, hippocampus, and substantia nigra, and that RAB39B was abundant in cortical and hippocampal neurons, as well as in dopaminergic neurons in the SNpc (Gao et al., 2020). Herein, we found that RAB39B was specifically expressed in neurons and comparably expressed in mouse hippocampus, cortex, midbrain, and cerebellum. Levels of Iba1 (a microglia marker) and GFAP (an astrocyte marker) in these brain regions were comparable between 2-month-old WT and *Rab39b* KO mice (Supplementary

Figures 5B and 8A), suggesting that loss of *Rab39b* has no effect on glial activation at this age. Since loss-of-function *RAB39B* mutations are associated with PD that has dramatic dopaminergic neuronal loss and dopamine dyshomeostasis during neurodegeneration (Masato et al., 2019; Shao and Le, 2019), we studied levels of dopamine and the dopaminergic neuronal marker tyrosine hydroxylase (TH) in the midbrain of 2-month-old *Rab39b* KO mice. Interestingly, we found that TH levels were increased and dopamine levels were decreased in *Rab39b* KO mouse midbrain compared to controls (Supplementary Figures 8A,B). One possible reason for this is that loss of *Rab39b* leads to dopamine level reduction, which causes a compensatory increase in dopaminergic neuron numbers. How RAB39B deficiency exactly affects dopaminergic neurons at this age and whether the effect changes during aging deserve further scrutiny.

In another recent study, Zhang *et al.* also generated *Rab39b* KO mice (in C57BL/6N background) using the CRISPR/Cas9 technique and described that these mice had cortical neurogenesis impairment, macrocephaly, and social memory and motor skill learning deficits reminiscent patient phenotypes (Zhang et al., 2020). While our current study confirmed phenotypic effect of *Rab39b* KO on motor skill learning and social memory, more importantly, we further investigated the detailed role of RAB39B in learning and memory. We found that *Rab39b* KO mice exhibited defects in short-term working memory, novel objection recognition memory, and spatial memory. Moreover, *Rab39b* KO mice exhibited impaired

synaptic plasticity and altered synaptic structure and PSD composition. Since cognitive impairment is a critical feature of ID as a formal diagnosis of ID is made only when the IQ is scored to be less than 70 (Vissers et al., 2016), our results strengthen the causal effect of *RAB39B* mutations in the etiology of XLID. The PI3K-AKT-mTOR pathway is an important biological process that regulates cell differentiation, proliferation, migration, and metabolism during development. Dysfunction of this pathway may cause various neurological disorders such as ASD, focal cortical dysplasia, etc. (Costa-Mattioli and Monteggia, 2013; Dibble and Cantley, 2015). Zhang *et al.* found that RAB39B could interact with PI3K components and loss of RAB39B promoted the PI3K-AKT-mTOR pathway, leading to neural progenitor cell (NPC) over-proliferation and macrocephaly in mice at an age of postnatal day 20 (Zhang et al., 2020). Similarly, we also found that RAB39B deficiency resulted in markedly increased levels of phosphorylated S6, indicating the activation of the PI3K-AKT-mTOR pathway. However, we did not observe obvious macrocephaly in our *Rab39b* KO mice at 2 months of age. This discrepancy may be attributed to differences between mouse genetic backgrounds in the current study (C57BL/6J) and in Zhang et al. (C57BL/6N). C57BL/6J but not C57BL/6N mice carry a loss-of-function mutation in the nicotinamide nucleotide transhydrogenase gene and this makes C57BL/6J mice more susceptible to diet-induced obesity than C57BL/6N mice (Nicholson et al., 2010). Therefore, different metabolism and possibly other different biological processes between the two mouse strains may interfere with the effect of *Rab39b*

deficiency on brain growth. The fact that not all patients carrying loss-of-function *RAB39B* mutations have macrocephaly (Supplementary Table 1) also suggest that loss of *Rab39b* does not necessarily lead to macrocephaly in mice; and other factors may coordinate with *Rab39b* deficiency for this abnormality development. Alternatively, this discrepancy may be caused by the different mouse ages studied. It was reported that mouse cortical structural growth had a near exponential growth rate initially but tapered off at postnatal days 15-20; and subsequently the growth rate kept at a steady state (Baloch et al., 2009). Therefore, it is possible that loss of *Rab39b* boosts brain growth dramatically during an early brain developmental stage and thus results in significant brain size difference between WT and *Rab39b* KO mice when the brain growth rate reaches the peak (e.g. at postnatal day 20). While after this stage, both WT and *Rab39b* KO mouse brain growth rates slow down and the effect of *Rab39b* deficiency on NPC over-proliferation also decreases as active NPC numbers drop during brain development, narrowing the gap between WT and *Rab39b* KO mouse brain sizes in adults.

Autophagy is a conserved mechanism for degrading unnecessary or abnormal cytoplasmic entities to maintain cellular homeostasis in response to stress. Autophagy dysregulation may result in neurodevelopmental as well as neurodegenerative diseases (Lee et al., 2013; Frake et al., 2015; Beltran et al., 2019; Lachance et al., 2019). Herein, we found that RAB39B deficiency resulted in elevated LC3B-II levels by impairing autophagic flux at

basal level; and this is consistent with the elevated PI3K-AKT-mTOR signaling upon loss of *Rab39b* since this mTOR signaling inhibits autophagy (Dibble and Cantley, 2015). Coincidently, the C9ORF72/WDR41/SMCR8/ATG101 complex acts as a GDP/GTP exchange factor for RAB39B and deficiency in components of this complex can also alter autophagy, implying that such alterations may be mediated by RAB39B (Sellier et al., 2016; Yang et al., 2016; Corbier and Sellier, 2017; Tang et al., 2020). Moreover, we found that treatment with the mTOR inhibitor rapamycin comparably stimulated autophagy in both control and RAB39B-deficient cells, suggesting that RAB39B deficiency impairs basal autophagic flux but fails to affect cellular response to autophagy simulation. Interestingly, we noticed that levels of p62, another commonly used autophagy marker were not changed in *Rab39b* KO mice compared to WT controls (Figure 4A and Supplementary Figure 6). Since p62 regulates diverse processes such as apoptosis and necroptosis and interacts with several signaling molecule that affect p62 transcriptional synthesis (Sahani et al., 2014; Sanchez-Martin et al., 2019), p62 may be dispensable for canonical autophagy in *Rab39b* KO mice; and this deserves further scrutiny.

Upregulation of autophagy has been found to reverse disease-like phenotypes in animal models of FCD, PD, AD, etc. (Lee et al., 2013; Frake et al., 2015; Zhao et al., 2019b). Herein, we also found that rapamycin treatment improved novel object recognition memory and rescued LTP deficits in *Rab39b* KO mice, suggesting that rapamycin may also alleviate

symptoms in XLID patients caused by *RAB39B* mutations.

AMPA receptors are the most common excitatory glutamate receptors in the brain, which are tetramers composed of four subunit types (GluA1-GluA4) and can be directly activated upon glutamate binding. AMPA receptors primarily mediate rapid electrophysiological responses to glutamate. NMDA receptors mainly exist in the postsynaptic membrane and are also tetramers composed of two GluN1 subunits together with either two GluN2 subunits or one GluN2 and one GluN3 subunits. NMDA receptor activation by glutamate requires either glycine or D-serine as a co-agonist, and is relatively slow and prolonged compared to AMPA receptor activation (Scannevin and Huganir, 2000; Hettinger et al., 2018; Hanada, 2020). A previous study found that RAB39B could mediate trafficking of the AMPA receptor subunit GluA2 and downregulation of RAB39B skewed AMPA receptor composition towards non-GluA2-containing calcium-permeable forms, affecting mouse hippocampal neuron synaptic activity (Mignogna et al., 2015). In the PSD of *C9orf72* KO mice whose RAB39B GDP/GTP exchange factor is impaired, levels of RAB39B were decreased, whereas levels of the AMPA receptor subunit GluA1 were increased (Xiao et al., 2019), implying that RAB39B may affect GluA1 trafficking. However, we found that in *Rab39b* KO mice, the PSD distribution of NMDA receptors rather than AMPA receptors were significantly decreased. The discrepancy between our and others' results may be attributed to the different cell and mouse lines used. Nevertheless, electrophysiological studies revealed decreased NMDA/AMPA ratios and

unaltered AMPA-eEPSC amplitudes in our *Rab39b* KO mice compared to WT controls, strengthening a functional deficiency of NMDA receptors rather than AMPA receptors in these mice. Moreover, rapamycin treatment partially rescued memory and LTP deficits without obviously affecting NDMA receptor distribution, therefore, the contribution of defective autophagy and NMDA receptors to synaptic functions and learning and memory in *Rab39b* KO mice may be executed through different mechanisms.

In summary, our study demonstrates that the neuron-specific protein RAB39B plays a crucial role in regulating learning and memory. Deletion of *Rab39b* leads to synaptic dysfunction and autophagy disturbance. Rapamycin treatment can partially rescue impaired memory and synaptic plasticity in *Rab39b* KO mice (Figure 6). These findings not only demonstrate the importance of RAB39B in learning and memory through modulating both autophagy and synapse formation, but also suggest that targeting autophagy holds potential for intervention of learning and memory deficits in patients carrying *RAB39B* mutations. In the current study we only carried out research in young mice. Since *RAB39B* mutations are also associated with the neurodegenerative disease PD, the age-dependent changes in *Rab39b* KO mice and whether these changes can be reversed by modulating autophagy and/or glutamate receptors warrant further scrutiny.

DATA AVAILABILITY STATEMENT

The raw data supporting the conclusions of this study will be made available by the authors to any qualified researcher.

AUTHOR CONTRIBUTIONS

M.N. and Y-w.Z. designed research. M.N., Z.W., X.L., X.F., Y.W., M.L., T.Y., P.Y., J.M., and Y.Z. conducted molecular, cellular and/or animal experiments. N.Z., Y.Gao, Z.C., and T.W. performed electrophysiological experiments. Y.Ge, Z.W., and Q.M. made intellectual contributions. M.N., H.X., and Y-w.Z. wrote the manuscript. Y-w.Z. supervised the project. All authors reviewed the manuscript.

FUNDING

This work was supported by grants from National Natural Science Foundation of China (81771377 and U1705285 to Y-w.Z.), National Key Research and Development Program of China (2018YFC2000400 and 2016YFC1305903 to Y-w.Z.), Fundamental Research Funds for the Central Universities (20720180049 to Y-w.Z.), and Students' Platform for Innovation

and Entrepreneurship Training Program of Xiamen University (2019Y1210 to Y.T.).

ACKNOWLEDGMENTS

We thank Lei Shi, Hao Sun, Yanfang Li, Dan Can, Hong Luo, and Xian Zhang for technical assistance.

SUPPLEMENTARY MATERIAL

The Supplementary Material for this study can be found at Frontiers' website.

REFERENCES

Acquarone, E., Argyrousi, E.K., van den Berg, M., Gulisano, W., Fa, M., Staniszewski, A., et al. (2019). Synaptic and memory dysfunction induced by tau oligomers is rescued by up-regulation of the nitric oxide cascade. *Mol. Neurodegener.* 14(1), 26. doi: 10.1186/s13024-019-0326-4.

Baloch, S., Verma, R., Huang, H., Khurd, P., Clark, S., Yarowsky, P., et al. (2009).

Quantification of Brain Maturation and Growth Patterns in C57BL/6J Mice via Computational Neuroanatomy of Diffusion Tensor Images. *Cerebral Cortex* 19(3), 675-687. doi: 10.1093/cercor/bhn112.

Beltran, S., Nassif, M., Vicencio, E., Arcos, J., Labrador, L., Cortes, B.I., et al. (2019). Network approach identifies Pacer as an autophagy protein involved in ALS pathogenesis. *Mol. Neurodegener.* 14(1), 14. doi: 10.1186/s13024-019-0313-9.

Cheng, H., Ma, Y., Ni, X., Jiang, M., Guo, L., Ying, K., et al. (2002). Isolation and characterization of a human novel RAB (RAB39B) gene. *Cytogenet. Genome Res.* 97(1-2), 72-75. doi: 10.1159/000064047.

Ciammola, A., Carrera, P., Di Fonzo, A., Sassone, J., Villa, R., Poletti, B., et al. (2017). X-linked Parkinsonism with Intellectual Disability caused by novel mutations and somatic mosaicism in RAB39B gene. *Parkinsonism Relat. Disord.* 44, 142-146. doi: 10.1016/j.parkreldis.2017.08.021.

Corbeel, L., and Freson, K. (2008). Rab proteins and Rab-associated proteins: major actors in the mechanism of protein-trafficking disorders. *Eur. J. Pediatr.* 167(7), 723-729. doi: 10.1007/s00431-008-0740-z.

Corbier, C., and Sellier, C. (2017). C9ORF72 is a GDP/GTP exchange factor for Rab8 and Rab39 and regulates autophagy. *Small GTPases* 8(3), 181-186. doi:

10.1080/21541248.2016.1212688.

Costa-Mattioli, M., and Monteggia, L.M. (2013). mTOR complexes in neurodevelopmental and neuropsychiatric disorders. *Nat. Neurosci.* 16(11), 1537-1543. doi: 10.1038/nn.3546.

Dibble, C.C., and Cantley, L.C. (2015). Regulation of mTORC1 by PI3K signaling. *Trends Cell Biol.* 25(9), 545-555. doi: 10.1016/j.tcb.2015.06.002.

Frake, R.A., Ricketts, T., Menzies, F.M., and Rubinsztein, D.C. (2015). Autophagy and neurodegeneration. *J. Clin. Invest.* 125(1), 65-74. doi: 10.1172/JCI73944.

Gao, Y., Wilson, G.R., Stephenson, S.E.M., Oulad-Abdelghani, M., Charlet-Berguerand, N., Bozaoglu, K., et al. (2020). Distribution of Parkinson's disease associated RAB39B in mouse brain tissue. *Mol. Brain* 13(1), 52. doi: 10.1186/s13041-020-00584-7.

Giannandrea, M., Bianchi, V., Mignogna, M.L., Sirri, A., Carrabino, S., D'Elia, E., et al. (2010). Mutations in the small GTPase gene RAB39B are responsible for X-linked mental retardation associated with autism, epilepsy, and macrocephaly. *Am. J. Hum. Genet.* 86(2), 185-195. doi: 10.1016/j.ajhg.2010.01.011.

Guldner, M., Schulte, C., Hauser, A.K., Gasser, T., and Brockmann, K. (2016). Broad clinical phenotype in Parkinsonism associated with a base pair deletion in RAB39B and additional POLG variant. *Parkinsonism Relat. Disord.* 31, 148-150. doi:

10.1016/j.parkreldis.2016.07.005.

Hanada, T. (2020). Ionotropic Glutamate Receptors in Epilepsy: A Review Focusing on AMPA and NMDA Receptors. *Biomolecules* 10(3), 464. doi: 10.3390/biom10030464.

Hettinger, J.C., Lee, H., Bu, G., Holtzman, D.M., and Cirrito, J.R. (2018). AMPA-ergic regulation of amyloid-beta levels in an Alzheimer's disease mouse model. *Mol. Neurodegener.* 13(1), 22. doi: 10.1186/s13024-018-0256-6.

Iwase, S., Berube, N.G., Zhou, Z., Kasri, N.N., Battaglioli, E., Scandaglia, M., et al. (2017). Epigenetic Etiology of Intellectual Disability. *J. Neurosci.* 37(45), 10773-10782. doi: 10.1523/JNEUROSCI.1840-17.2017.

Kimura, S., Noda, T., and Yoshimori, T. (2007). Dissection of the autophagosome maturation process by a novel reporter protein, tandem fluorescent-tagged LC3. *Autophagy* 3(5), 452-460. doi: DOI 10.4161/auto.4451.

Lachance, V., Wang, Q., Sweet, E., Choi, I., Cai, C.Z., Zhuang, X.X., et al. (2019). Autophagy protein NRBF2 has reduced expression in Alzheimer's brains and modulates memory and amyloid-beta homeostasis in mice. *Mol. Neurodegener.* 14(1), 43. doi: 10.1186/s13024-019-0342-4.

Lee, K.M., Hwang, S.K., and Lee, J.A. (2013). Neuronal autophagy and neurodevelopmental disorders. *Exp. Neurobiol.* 22(3), 133-142. doi: 10.5607/en.2013.22.3.133.

Lesage, S., Bras, J., Cormier-Dequaire, F., Condroyer, C., Nicolas, A., Darwent, L., et al. (2016). Loss-of-function mutations in RAB39B are associated with typical early-onset Parkinson disease. *Neurol. Genet.* 1(1)**,** e9. doi: 10.1212/NXG.0000000000000009.

Masato, A., Plotegher, N., Boassa, D., and Bubacco, L. (2019). Impaired dopamine metabolism in Parkinson's disease pathogenesis. *Mol. Neurodegener.* 14(1)**,** 35. doi: 10.1186/s13024-019-0332-6.

Mata, I.F., Jang, Y., Kim, C.H., Hanna, D.S., Dorschner, M.O., Samii, A., et al. (2015). The RAB39B p.G192R mutation causes X-linked dominant Parkinson's disease. *Mol. Neurodegener.* 10**,** 50. doi: 10.1186/s13024-015-0045-4.

Mignogna, M.L., Giannandrea, M., Gurgone, A., Fanelli, F., Raimondi, F., Mapelli, L., et al. (2015). The intellectual disability protein RAB39B selectively regulates GluA2 trafficking to determine synaptic AMPAR composition. *Nat. Commun.* 6**,** 6504. doi: 10.1038/ncomms7504.

Nicholson, A., Reifsnyder, P.C., Malcolm, R.D., Lucas, C.A., MacGregor, G.R., Zhang, W.D., et al. (2010). Diet-induced Obesity in Two C57BL/6 Substrains With Intact or Mutant Nicotinamide Nucleotide Transhydrogenase (Nnt) Gene. *Obesity* 18(10)**,** 1902-1905. doi: 10.1038/oby.2009.477.

Peng, J.P., Liu, F., Xie, H., and Chen, X.L. (2017). The pathogenicity of genomic/genetic

variant of X-chromosomal genes in males with intellectual disability. *Yi Chuan* 39(6), 455-468. doi: 10.16288/j.yczz.16-407.

Sahani, M.H., Itakura, E., and Mizushima, N. (2014). Expression of the autophagy substrate SQSTM1/p62 is restored during prolonged starvation depending on transcriptional upregulation and autophagy-derived amino acids. *Autophagy* 10(3), 431-441. doi: 10.4161/auto.27344.

Sanchez-Martin, P., Saito, T., and Komatsu, M. (2019). p62/SQSTM1: 'Jack of all trades' in health and cancer. *FEBS J.* 286(1), 8-23. doi: 10.1111/febs.14712.

Santoro, C., Giugliano, T., Bernardo, P., Palladino, F., Torella, A., Del Vecchio Blanco, F., et al. (2020). A novel RAB39B mutation and concurrent de novo NF1 mutation in a boy with neurofibromatosis type 1, intellectual disability, and autism: a case report. *BMC Neurol.* 20(1), 327. doi: 10.1186/s12883-020-01911-0.

Scannevin, R.H., and Huganir, R.L. (2000). Postsynaptic organization and regulation of excitatory synapses. *Nat. Rev. Neurosci.* 1(2), 133-141. doi: 10.1038/35039075.

Sellier, C., Campanari, M.L., Julie Corbier, C., Gaucherot, A., Kolb-Cheynel, I., Oulad-Abdelghani, M., et al. (2016). Loss of C9ORF72 impairs autophagy and synergizes with polyQ Ataxin-2 to induce motor neuron dysfunction and cell death. *EMBO J.* 35(12), 1276-1297. doi: 10.15252/embj.201593350.

Shao, Y., and Le, W. (2019). Recent advances and perspectives of metabolomics-based investigations in Parkinson's disease. *Mol. Neurodegener.* 14(1), 3. doi: 10.1186/s13024-018-0304-2.

Shi, C.H., Zhang, S.Y., Yang, Z.H., Yang, J., Shang, D.D., Mao, C.Y., et al. (2016). A novel RAB39B gene mutation in X-linked juvenile parkinsonism with basal ganglia calcification. *Mov. Disord.* 31(12), 1905-1909. doi: 10.1002/mds.26828.

Tang, X., Toro, A., T, G.S., Gao, J., Chalk, J., Oskarsson, B., et al. (2020). Divergence, Convergence, and Therapeutic Implications: A Cell Biology Perspective of C9ORF72-ALS/FTD. *Mol. Neurodegener.* 15(1), 34. doi: 10.1186/s13024-020-00383-7.

Ung, D.C., Iacono, G., Meziane, H., Blanchard, E., Papon, M.A., Selten, M., et al. (2018). Ptchd1 deficiency induces excitatory synaptic and cognitive dysfunctions in mouse. *Mol. Psychiatry* 23(5), 1356-1367. doi: 10.1038/mp.2017.39.

Vissers, L.E., Gilissen, C., and Veltman, J.A. (2016). Genetic studies in intellectual disability and related disorders. *Nat Rev Genet* 17(1), 9-18. doi: 10.1038/nrg3999.

Wang, X., Zhao, Y., Zhang, X., Badie, H., Zhou, Y., Mu, Y., et al. (2013). Loss of sorting nexin 27 contributes to excitatory synaptic dysfunction by modulating glutamate receptor recycling in Down's syndrome. *Nat. Med.* 19(4), 473-480. doi: nm.3117 [pii] 10.1038/nm.3117.

Wen, L., Lu, Y.S., Zhu, X.H., Li, X.M., Woo, R.S., Chen, Y.J., et al. (2010). Neuregulin 1 regulates pyramidal neuron activity via ErbB4 in parvalbumin-positive interneurons. *Proc. Natl. Acad. Sci. U. S. A.* 107(3), 1211-1216. doi: 10.1073/pnas.0910302107.

Wen, L., Tang, F.L., Hong, Y., Luo, S.W., Wang, C.L., He, W., et al. (2011). VPS35 haploinsufficiency increases Alzheimer's disease neuropathology. *J. Cell Biol.* 195(5), 765-779. doi: jcb.201105109 [pii] 10.1083/jcb.201105109.

Woodbury-Smith, M., Deneault, E., Yuen, R.K.C., Walker, S., Zarrei, M., Pellecchia, G., et al. (2017). Mutations in RAB39B in individuals with intellectual disability, autism spectrum disorder, and macrocephaly. *Mol. Autism* 8, 59. doi: 10.1186/s13229-017-0175-3.

Xiao, S., McKeever, P.M., Lau, A., and Robertson, J. (2019). Synaptic localization of C9orf72 regulates post-synaptic glutamate receptor 1 levels. *Acta Neuropathol. Commun.* 7(1), 161. doi: 10.1186/s40478-019-0812-5.

Yang, M., Liang, C., Swaminathan, K., Herrlinger, S., Lai, F., Shiekhattar, R., et al. (2016). A C9ORF72/SMCR8-containing complex regulates ULK1 and plays a dual role in autophagy. *Sci. Adv.* 2(9), e1601167. doi: 10.1126/sciadv.1601167.

Zeng, F., Ma, X., Zhu, L., Xu, Q., Zeng, Y., Gao, Y., et al. (2019). The deubiquitinase USP6 affects memory and synaptic plasticity through modulating NMDA receptor stability.

PLoS Biol. 17(12), e3000525. doi: 10.1371/journal.pbio.3000525.

Zhang, W., Ma, L., Yang, M., Shao, Q., Xu, J., Lu, Z., et al. (2020). Cerebral organoid and mouse models reveal a RAB39b-PI3K-mTOR pathway-dependent dysregulation of cortical development leading to macrocephaly/autism phenotypes. *Genes Dev.* 34(7-8), 580-597. doi: 10.1101/gad.332494.119.

Zhao, D., Meng, J., Zhao, Y., Huo, Y., Liu, Y., Zheng, N., et al. (2019a). RPS23RG1 Is Required for Synaptic Integrity and Rescues Alzheimer's Disease-Associated Cognitive Deficits. *Biol. Psychiatry* 86(3), 171-184. doi: 10.1016/j.biopsych.2018.08.009.

Zhao, S., Li, Z., Zhang, M., Zhang, L., Zheng, H., Ning, J., et al. (2019b). A brain somatic RHEB doublet mutation causes focal cortical dysplasia type II. *Exp. Mol. Med.* 51(7), 84. doi: 10.1038/s12276-019-0277-4.

Zhou, J., Blundell, J., Ogawa, S., Kwon, C.H., Zhang, W., Sinton, C., et al. (2009). Pharmacological inhibition of mTORC1 suppresses anatomical, cellular, and behavioral abnormalities in neural-specific Pten knock-out mice. *J. Neurosci.* 29(6), 1773-1783. doi: 10.1523/JNEUROSCI.5685-08.2009.

Conflict of Interest: The authors declare that the research was conducted in the absence of any commercial or financial relationships that could be construed as a potential conflict of interest.

FIGURE LEGENDS

FIGURE 1 Characterization of *Rab39b* KO mice. **(A)** Sequence alignment of RAB39B proteins of wild type (213 amino acids) and knockout (KO, 88 amino acids) mice. Conserved residues are highlighted in green. **(B)** RAB39B proteins in brain samples of wild type (*Rab39b*$^{+/+}$ and *Rab39b*$^{+/Y}$), heterozygous female (*Rab39b*$^{+/-}$), homozygous female (*Rab39b*$^{-/-}$), and hemizygous male (*Rab39b*$^{-/Y}$) KO mice from same litters were detected by western blot. **(C)** *Rab39b*$^{+/-}$ female and *Rab39b*$^{-/Y}$ male mice were crossed. Numbers of males and females, as well as different genotypes of the offspring were counted for studying if they follow a Mendelian frequency. **(D)** Observation of the overall body and brain (before and after perfusion) morphologies of *Rab39b* KO mice and their wild type (WT) littermate controls. **(E)** Comparison of body weight, brain weight and the ratio of brain/body weight between *Rab39b* KO mice and their WT littermate controls at 2 months of age. Data represent mean ± SEM, n = 7 for each group, ns: not significant, $^{*}p < 0.05$, $^{**}p < 0.01$, Mann-Whitney test. **(F)** Cortical and hippocampal regions of WT and *Rab39b* KO mice at 2 months of age were immunostained with an anti-NeuN antibody (red) and stained with DAPI (blue), and observed under a confocal microscope. Scale bar, 500 μm.

FIGURE 2 *Rab39b* KO mice exhibit altered behavioral phenotypes. 2-month-old *Rab39b* KO

mice and WT littermate control mice were subjected to various behavioral tests. **(A)** Mice were studied for their time staying in the open arm and total travel distance in high elevated plus maze tests. **(B)** In novel object recognition tests, mice were evaluated for their recognition of two identical objects A and B on day 1 and identification of a novel object C on day 2. **(C)** Mice were tested for their spontaneous alternation in T maze tests. **(D)** Mice were analyzed for their escape latency in Morris Water Maze tests within a 5-day training period. On the 6th day, mice were assayed for time spent in the target and the other three quadrants. **(E)** In three-chamber social interaction tests, mouse sociability was studied by comparing their interactions with a stranger mouse (stranger 1) and with an empty cage. Social novelty was studied by comparing their interactions with the familiar mouse (stranger 1) and with a new stranger mouse (stranger 2). **(F)** Mice were tested for their time staying on a rotarod three times a day for three consecutive days. Data represent mean ± SEM, $n = 13$ for each group. For comparisons between WT and KO in **(A-F)**, ns: not significant, $^{*}p < 0.05$, $^{**}p < 0.01$, $^{***}p < 0.001$, $^{****}p < 0.0001$, Mann-Whitney test. For comparisons between rotarod staying times of other trials and rotarod staying time of the first trial within WT, $^{\#}p < 0.05$, $^{\#\#}p < 0.01$, $^{\#\#\#}p < 0.001$, Mann-Whitney test.

FIGURE 3 *Rab39b* KO mice exhibit defects in synaptic function and structure and PSD

composition. **(A)** Changes of eEPSCs were recorded in the CA1 region when increased stimulations were given in the CA3 region of WT and *Rab39b* KO mice. Input-output curves were subjected to comparison. Data represent mean ± SEM, $n = 6$ slices from 4 mice per group, $^{****}p < 0.0001$, repeated measures ANOVA. **(B)** Paired-pulse ratios of eEPSCs in the CA1 pyramidal neurons were subjected to comparison. Data represent mean ± SEM, $n = 6$ slices from 4 mice per group, ns: not significant, one-way ANOVA followed with Dunnett's test. **(C)** LTP was induced by a two-train (100 Hz, 30 sec interval) high frequency stimulation (HFS) in the CA3 region. The left panel shows representative fEPSP recording traces from 20 min before to 60 min after HFS in the CA1 region. The right panel shows comparisons of mean potentiation from the fEPSP slopes calculated between 0–10 min and 50–60 min after HFS. Data represent mean ± SEM, $n = 6$ slices from 4 mice per group, $^{**}p < 0.01$, Mann-Whitney test. **(D)** AMPA receptor- and NMDA receptor-mediated EPSCs were recorded from same hippocampal CA1 neurons and NMDA/AMPA receptor response ratios were calculated for comparison. Data represent mean ± SEM, $n = 6$ cells per group, $^{*}p < 0.05$, Mann-Whitney test. **(E)** AMPA-eEPSCs were recorded in hippocampal CA1 neurons at different stimulus intensities for comparison. Data represent mean ± SEM, $n = 7$ cells per group, $^{*}p < 0.05$, repeated measures ANOVA. **(F)** Cortical and hippocampal regions of WT and *Rab39b* KO mice were subjected to Golgi staining and microscopy. Scale bar, 5 µm. Apical distal spine numbers of neurons in cortical V1/V2 and hippocampal CA1 regions were quantified

respectively for comparison. Data represent mean ± SEM, $n = 15$ neurons from 3 mice per group, $^{*}p < 0.05$, $^{**}p < 0.01$, Mann-Whitney test. **(G)** Cortical V1/V2 and hippocampal CA1 regions of WT and *Rab39b* KO mice were subjected to electron microscopy. Scale bar, 100 nm. PSD length and width were quantified for comparison. Data represent mean ± SEM, $n = 66$ synapses from 3 mice per group, $^{****}p < 0.0001$, two-tailed Student's t test. **(H)** Equal quantities of brain samples of WT and *Rab39b* KO mice were fractionated to acquire total lysates, synaptosomal (Syn) fractions, and PSD fractions. Samples were subjected to western blot to detect the proteins indicated. Levels of proteins indicated were quantified by densitometry, normalized to those of β-actin, and compared to respective WT controls (set to one arbitrary units, A.U.). Data represent mean ± SEM, $n = 5$ for each group, ns: not significant, $^{**}p < 0.01$, Mann-Whitney test.

FIGURE 4 RAB39B deficiency impairs autophagy. **(A)** Equal protein quantities of hippocampus lysates derived from WT and *Rab39b* KO mice were subjected to western blot for the proteins indicated. Levels of p62, LC3B-II and S6 phosphorylated at S240/244 (p-S6) were quantified by densitometry and normalized to those of β-actin or S6, respectively, and compared to WT controls (set to one arbitrary units, A.U.). Data represent mean ± SEM, $n = 6$ for each group, ns: not significant, $^{*}p < 0.05$, $^{**}p < 0.01$, Mann-Whitney test. **(B)** N2a cells

were transfected with scrambled siRNA (ns) or an siRNA targeting *Rab39b* (si*Rab39b*) for 60 h. After RNA extraction and reverse transcription, *Rab39b* mRNA levels were detected by qRT-PCR, normalized to those of β-actin, and compared to ns controls (set to one A.U.). Data represent mean ± SEM, $n = 4$ for each group, $^{*}p < 0.05$, Mann-Whitney test. **(C)** N2a cells were transfected with ns or *siRab39b* for 60 h. Cells were then treated with 250 nM rapamycin for 0 or 30 min. Cells lysates were subjected to western blot for the proteins indicated. LC3B-II and p-S6 levels were quantified for comparison to respective ns controls (set to one A.U.). Data represent mean ± SEM, $n = 5$ for each group, ns: not significant, $^{**}p < 0.01$, Mann-Whitney test. **(D)** N2a cells were first transfected with ns or *siRab39b* for 36 h and then transfected with RFP-GFP-LC3B. After another 24 h, cells were treated with 250 nM rapamycin for 0 or 30 min. Images were acquired by confocal microscopy. Red and green colors indicate RFP and GFP, respectively. The siRNA was depicted in white. Scale bar, 10 μm. Data represent mean ± SEM, n= 21 from three independent experiments for each group, ns: not significant, $^{***}p$<0.001, $^{****}p$<0.0001, two-tailed Student's t test.

FIGURE 5 Rapamycin treatment partially rescues memory and LTP defects in *Rab39b* KO mice. **(A)** The workflow for rapamycin treatment and subsequent analysis. **(B)** Equal protein quantities of hippocampus lysates derived from *Rab39b* KO mice treated with rapamycin or

vehicle were subjected to western blot for the proteins indicated. LC3B-II and phosphorylated S6 (p-S6) levels were quantified by densitometry, normalized to those of β-actin and S6, respectively, and compared to controls (set to one arbitrary units, A.U.). Data represent mean ± SEM, $n = 7$ for vehicle group, $n = 6$ for rapamycin group, $^{*}p<0.05$, $^{**}p<0.01$, Mann-Whitney test. **(C)** Treated *Rab39b* KO mice were subjected to novel object recognition tests to study their recognition of two identical objects A and B on day 1 and recognization of a novel object C on day 2. Data represent mean ± SEM, $n = 9$ for each group, ns: not significant, $^{**}p < 0.01$, Mann-Whitney test. **(D)** LTP was induced by a two-train (100 Hz, 30 sec interval) high frequency stimulation (HFS) in the CA3 region of mice. The upper panel is representative fEPSP recording traces from 20 min before to 60 min after HFS in the CA1 region. The lower panel shows comparisons of mean potentiation from the fEPSP slopes calculated between 0–10 min and 50–60 min after HFS. Data represent mean ± SEM, $n = 8$ slices from 4 mice per group, ns: not significant, $^{**}p < 0.01$, Mann-Whitney test. **(E)** Equal quantities of brain samples of treated *Rab39b* KO mice were fractionated to acquire total lysates, synaptosomal (Syn) fractions, and PSD fractions. Samples were subjected to western blot to detect the proteins indicated. GluN1, GluN2A, and GluN2B levels were quantified by densitometry, normalized to those of β-actin, and compared to respective controls (set to one A.U.). Data represent mean ± SEM, $n = 6$ for each group, ns: not significant, $^{*}p < 0.05$, Mann-Whitney test.

FIGURE 6 Scheme of RAB39B deficiency induced synaptic and learning and memory impairment. Deletion of *Rab39b* results in defective synaptic structure and function and PSD distribution of NMDA receptors, as well as increased mTOR signaling and compromised autophagy flux. Rapamycin treatment stimulates autophagy through inhibiting mTOR and partially rescues impaired memory and synaptic plasticity in *Rab39b* KO mice.

RAB39B deficiency impairs learning and memory partially through compromising autophagy

Supplementary Materials

Supplementary MATERIALS AND METHODS

Rab39b KO mouse generation and genotyping

Mouse *Rab39b* gene has two exons, with the protein coding sequence (CDS) spanning both exons (Figure S2a). A TALEN-mediated strategy to target *Rab39b* exon 1 was utilized to generate *Rab39b* KO mice, with the service provided by Cyagen Biosciences Inc. The left (targeting 5'-TTCACCGAGGGCCGCTTT -3') and right (targeting 5'-TCTACCCCCACGGTGGGA-3') TALEN arms were designed and constructed (Figure S2b). Constructs were transcribed *in vitro*, and TALEN mRNAs were injected into fertilized C57BL/6J mouse eggs to generate *Rab39b* KO animals. For mouse genotyping, genomic DNA was extracted from mouse tails by dissolving in 0.02 M NaOH at 98℃ for 30 min and then neutralizing with 1 M Tris-HCl (pH 8.0). PCR primers used for genotyping were: 5'-CGATCTCCACCAAACGGGAG-3' and 5'-GTGGGCAAGTCCTGCCTGAT-3'. An 103 bp

fragment and an 101 bp fragment were PCR-amplified from wild type and *Rab39b* KO alleles, respectively, and resolved using a 15% Native-PAGE.

Western blot

Mouse tissue samples were lysed in RIPA lysis buffer [150 mM NaCl, 25 mM Tris–HCl, pH 8.0, 0.5% (wt/vol) sodium deoxycholate, 0.05% (wt/vol) sodium dodecyl sulfate, and 1% (vol/vol) Nonidet P-40] supplemented with protease and phosphatase inhibitor cocktails for 40 min. Cells were lysed in TNEN lysis buffer [50 mM Tris–HCl, 150 mM NaCl, pH 8.0, 2 mM EDTA, and 1% (vol/vol) Nonidet P-40] supplemented with protease and phosphatase inhibitor cocktails for 30 min. Equal amounts of protein lysates were resolved using SDS-polyacrylamide gel electrophoresis, transferred to polyvinylidene difluoride membranes (Merck Millipore), probed with primary antibodies and then secondary antibodies as indicated, and detected by chemiluminescence.

Mouse behavioral tests

Nine cohorts of mice were subjected to behavioral experiments with the following order: open field tests, novel object recognition tests, high elevated plus maze tests, T-maze tests, three-chamber social interaction tests, Morris water maze tests, rotarod tests, and hanging tests.

Open field tests were used to detect locomotor activity and anxiety behavior of mice. The open field box is a rectangular box (40 cm (L) x 40 cm (W) x 40 cm (H)). The mouse

exploratory activity was recorded for 10 min by Smart 3.0 video tracking system (Panlab). Total distance travelled in the arena and time spent in the center were measured for comparison.

High elevated plus maze tests were used to detect mouse anxiety. The maze is composed of two open arms and two closed arms forming a “+” shape (15 cm (L) x 6 cm (W)). Mice were placed in the center of the maze facing to an open arm. The exploratory activity was recorded for 5 min. Mouse exploring time in open arms was recorded by Smart 3.0 camera system for comparison.

T-maze tests were used to measure spontaneous alternation behaviors that indicate short-term working memory. The maze has three equally spaced arms forming a “T” shape (30cm (L) × 6cm (W) × 15cm (H)). Mice were placed in the center of the maze and their exploratory activity was recorded for 5 min. Sequence of arm entries and total number of arms entered were recorded by Smart 3.0.

Novel object recognition tests contain habituation, training and testing phases. Mice were first allowed to habituate to an open field box (40 cm (L) x 40 cm (W) x 40 cm (H)) for 10 min. In the training phase, mice were allowed to explore the open field box with two identical objects (A and B) for 10 min. After 24 h, object B was replaced with a novel object (C) and mice were tested for their exploring times to objects A and C for 10 min. Mouse exploration time to each object was counted for comparison.

Morris water maze is a circular tank (120 cm in diameter) filled with tap water at a temperature of 22±2°C and contains an underwater platform. Walls surrounding the tank are taped with different shapes of pictures that serve as visual reference cues. The experiment was divided into two parts. In the training part, mice were placed into water to let them swim, and the time mice used to find and climb onto the platform was recorded. The maximum time mice allowed to swim were 60 s. If mice could not find the platform within 60 s, they were guided to the platform and allowed to stay on the platform for 5 s. Mice were trained for five days and on each day, mice were trained for four times by placing into the water from four different positions (N, S, W, and E). In the testing part, the underwater platform was removed on the 6th day and mice were placed into water to let them search the underwater platform. The percentage time mice spent in each of the four quadrants was recorded by Smart 3.0 for comparison.

Three-chamber social interaction tests were used to detect the social interaction behavior of mice. The social box is a rectangle box (60 cm (L) x 42 cm (W) x 22 cm (H)) and divided into three equal chambers (left, middle, and right) along its long side by two transparent plastic plates. There is a door on the plate to allow mice move between chambers. There is a small cage in each of the two side chambers. Firstly, mice were placed in the middle chamber with both cages empty to let mice explore the whole box freely for 5 min. After a stranger mouse was put into the cage in the left chamber, tested mice were allowed to continue exploring the

whole box for another 10 min. Finally, another stranger mouse was put into the cage in the right chamber, and tested mice were allowed to continue exploring the whole box for another 10 min. Mouse exploring time to each cage at different stages were recorded by Smart 3.0 for comparison.

Rotarod tests were used to measure motor and balance ability of mice. Mice were trained on the rotarod at 4 rpm until their latency time to fall was longer than 30 sec before testing. In the testing phase, mice were placed on the rotarod starting at 4 rpm and the rotarod was accelerated at a frequency of 5 rpm/min. The maximum time for mice to stay on the rod was 120 s. Mice were given three tests a day for three consecutive days. The latency time to fall was recorded by PenLab.

Hanging tests were used to detect the griping power of mice. Mice were made to grab and hang on a square wire mesh placed at a height of 50 cm above the ground, and the maximum time for mice to hang upside down was 180 s. The latency time to fall was recorded for comparison.

Electrophysiology

Ice-cold solution (64 mM NaCl, 2.5 mM KCl, 10 mM glucose, 1.25 mM NaH_2PO_4, 10 mM $MgSO_4$, 26 mM $NaHCO_3$, 120 mM sucrose, and 0.5 mM $CaCl_2$) was prepared and frozen in -80 °C 1 h before experiments. Mice were anesthetized and mouse brains were quickly

dissected by scissors and cut into 400 μm thick coronal slices by vibrating microtome (Leica VT1200S) in ice-cold solution aerated with 95% O_2/5% CO_2. Slices were placed into artificial cerebrospinal fluid (aCSF) containing 126 mM NaCl, 3.5 mM KCl, 1.25 mM NaH_2PO_4, 1.3 mM $MgSO_4$, 2.5 mM $CaCl_2$, 26 mM $NaHCO_3$, and 10 mM glucose. Slices were recovered for 1 h at 34°C and then at room temperature for an additional 2-8 h in aCSF. All solutions were saturated with 95% O_2/5% CO_2. *Evoked-excitatory* postsynaptic current (eEPSC) amplitudes were recorded in the CA1 region when different stimulus intensities (0.2, 0.4, 0.6, 0.8, 1.0, 1.2, 1.4, 1.6, 1.8, and 2.0 mA) were applied in the CA3 region. Paired-pulse ratio was measured by different inter-pulse intervals (10, 20, 50, 100, and 200 ms) in the CA3 region. LTP in the CA1 was recorded after the CA3 area was induced by two trains of 100-HZ stimuli (with 30 s interval).

Evoked EPSCs (eEPSCs) were recorded by stimulating the schaffer collateral pathway with a two-concentric bipolar stimulating electrode (FHC, Inc) positioned ~250 μm from CA1 pyramidal neurons. 5 mM lidocaine N-ethylchloride (QX-314) was added in the pipette. AMPA and NMDA receptor-mediated eEPSCs were recorded at a holding potential of -70 mV and +40 mV, respectively. 100 μM picrotoxin was added in the extracellular solution. The resistance of pipettes was 5-8 MΩ. Data were filtered at 0.5 kHz and sampled at 10 kHz, and acquired with a patch-clamp amplifier (Multiclamp 700B, Molecular Devices) and analyzed using pClamp 10.6 software (Molecular Devices). NMDA/AMPA receptor response ratios

were calculated for comparison. Alternatively, AMPA receptor-mediated eEPSCs was recorded at a holding potential of -70 mV and stimulated with increased intensities at 10, 30, 60, 90, 120, 150, 180, 210, 240, 270 and 300 μA. 100μM picrotoxin and 50 μM D-AP5 was added in the extracellular solution.

Electron microscope analysis

After anaesthetized, mice were perfused with saline followed by fixative solution (4%(wt/vol) paraformaldehyde and 2.5%(vol/vol) glutaraldehyde in 0.1 M phosphate buffer, pH 7.4). of Mouse cortical V1/V2 and hippocampal CA1 regions were dissected and stored with a fixative solution supplemented with 4%(wt/vol) paraformaldehyde and 1%(vol/vol) glutaraldehyde in 0.1 M phosphate buffer (pH 7.2) for 48 h. Tissues were washed in phosphate-buffered saline, followed by incubating with 2%(wt/vol) osmium tetroxide for 1 h and dehydrating in a graded series of ethanol solutions. Samples were embedded in Epon resin. After polymerization, ultra-thin sections were cut, stained with 2.5%(wt/vol) uranyl acetate and 1%(wt/vol) lead citrate, and deposited on electron microscope grids for examination under a JEOL 1011 (Tokyo, Japan) transmission electron microscope.

Preparation of synaptosomal and PSD fractions

Mouse tissues were dissected and homogenized in cold sucrose buffer (0.32 M sucrose and 25 mM HEPES, pH 7.4). Homogenates were centrifuged at 1,400 g for 10 min to separate the

supernatant (S1; total). The S1 fraction was centrifuged at 10,000 g for 12 min to separate the supernatant (S2; light membrane and cytosolic fraction) and the precipitation (P2; crude synaptosomal fraction). The P2 fraction was washed with sucrose buffer and re-suspended in cold HBS buffer (25 mM HEPES, pH 7.4, and 150 mM NaCl) to get the synaptosomal (Syn) fraction. The synaptosomal fraction was re-suspended in sucrose buffer with 1%(vol/vol) Triton X-100 at 4°C for 40 min, followed by 40,000 g centrifugation for 30 min to obtain the precipitation (PSD fraction).

Dopamine ELISA Assay

Dopamine concentrations in mouse midbrain were quantified by ELISA using Mouse DA ELISA Kits (Meimian, Cat#MM-0626M1), following the manufacturer's instructions.

Supplementary FIGURE LEGENDS

Supplementary FIGURE 1 Mouse RAB39B expression pattern. **(A)** RAB39B protein levels in various tissues of 2-month-old wild type C57BL/6J mice were detected by western blot. **(B)** RAB39B protein levels in different brain tissues of 2-month-old wild type C57BL/6J mice were detected by western blot, quantified by densitometry, and normalized to those of β-actin for comparison. Relative RAB39B levels in cortex were set to one arbitrary units (A.U.). Data represent mean ± SEM, $n = 4$, ns: not significant, Mann-Whitney test. **(C)** RAB39B in mouse primary neurons, microglia, and astrocytes were detected by western blot, quantified by densitometry, and normalized to those of β-actin for comparison. Relative RAB39B levels in neurons were set to one A.U. Data represent mean ± SEM, $n = 4$, $^{*}p<0.05$, Mann-Whitney test. NeuN, Iba-1, and GFAP were detected to evaluate the purity of neurons, microglia, and astrocytes, respectively. **(D)** RAB39B, GluN1, and synapsin 1 (SYN1) proteins in brain lysates of C57BL/6J wild type mice at different postnatal days were detected by western blot. RAB39B levels were quantified by densitometry and normalized to those of β-actin for comparison. Relative RAB39B levels at P0 were set to one A.U. Data represent mean ± SEM, $n = 4$, ns: not significant, $^{*}p < 0.05$, Mann-Whitney test.

Supplementary FIGURE 2 Alignment of human and mouse RAB39B protein sequences. A different amino acid site is highlighted in green.

Supplementary FIGURE 3 Generation of *Rab39b* knockout (KO) mice. **(A)** Scheme of the mouse *Rab39b* gene. *Rab39b* has two exons and its protein coding sequence (CDS) spans both exons (225-866, nomenclature according to National Center for Biotechnology Information Reference Sequence: NM_175122). **(B)** Two TALEN spacers (TALEN-L and TALEN-R) designed to integrate mutations into *Rab39b* exon 1 are shown (in green). The two nucleotides "GT" (CDS sites 106-107) deleted in the generated mouse line are indicated in red. **(C)** Sequencing the "GT" deletion sites in wild type ($Rab39b^{+/+}$ or $Rab39b^{+/Y}$), heterozygous female ($Rab39b^{+/-}$), and homozygous female ($Rab39b^{-/-}$) or hemizygous ($Rab39b^{-/Y}$) KO mice. **(D)** Genotyping of wild type (WT), heterozygous female, homozygous female, and hemizygous male *Rab39b* KO mice. Amplified PCR products spanning the targeted mutation sites were subjected to SDS-PAGE analysis to resolve WT and mutant (Mut) fragments. **(E)** RNAs of WT and *Rab39b* KO mouse brain were extracted and reverse transcribed. *Rab39b* mRNA levels were detected by qRT-PCR and normalized to those of β-actin for comparison (WT controls were set to one arbitrary units, A.U.). Data represent mean ± SEM, $n = 4$ for

each group, $^{*}p < 0.05$, Mann-Whitney test. **(F)** The fluorescence intensity of NeuN staining in cortical and hippocampal regions in Figure 1f were measured by ImageJ and compared to respective WT controls (set to one A.U.). Data represent mean ± SEM, n= 5 for each group, ns: not significant, Mann-Whitney test.

Supplementary FIGURE 4 Loss of *Rab39b* has no effect on mouse locomotor activity and muscle strength. **(A)** WT and *Rab39b* KO mice were analyzed for their mean moving speed, total travel distance, and duration in the center in open field tests. **(B)** WT and *Rab39b* KO mice were analyzed for their total travel distance and mean swimming speed in water maze tests. **(C)** WT and *Rab39b* KO mice were studied for their hanging time and impulses in four-limb hanging tests. Data represent mean ± SEM, n = 13 for each group, ns: not significant, Mann-Whitney test.

Supplementary FIGURE 5 Loss of *Rab39b* has no effect on synaptic vesicle numbers and total levels of synapse-related proteins. **(A)** Synaptic vesicle numbers in Figure 3G were counted for comparison. Data represent mean ± SEM, n = 15 neurons from 3 mice per group, ns: not significant, Mann-Whitney test. **(B)** Equal protein quantities of cortex or hippocampus

lysates derived from WT and *Rab39b* KO mice were subjected to western blot to study the proteins indicated.

Supplementary FIGURE 6 RAB39B deficiency impairs autophagy. Equal protein quantities of cortex and midbrain lysates derived from WT and *Rab39b* KO mice were subjected to western blot for the proteins indicated. Levels of p62, LC3B-II and S6 phosphorylated at S240/244 (p-S6) were quantified by densitometry and normalized to those of β-actin or S6, respectively, and compared to WT controls (set to one arbitrary units, A.U.). Data represent mean ± SEM, $n = 4$ for each group, $^{*}p < 0.05$, Mann-Whitney test.

Supplementary FIGURE 7 Rapamycin treatment does not affect locomotor activity, short-term working memory, or reduced anxiety in *Rab39b* KO mice. **(A-C)** *Rab39b* KO mice treated with DMSO vehicle or rapamycin were compared for their total travel distance and duration in the center in open field tests **(A)**, their spontaneous alternation in T maze tests **(B)**, and their time staying in the open arm and total travel distance in high elevated plus maze tests **(C)**. Data represent mean ± SEM, $n = 9$ in open field and T maze tests for each group, $n = 7$ in high elevated plus maze tests for each group, ns: not significant, Mann-Whitney test.

Supplementary FIGURE 8 RAB39B deficiency affects tyrosine hydrolysase (TH) and dopamine levels. **(A)** Equal protein quantities of midbrain lysates derived from WT and *Rab39b* KO mice were subjected to western blot for the proteins indicated. Levels of TH were quantified by densitometry and normalized to those of β-actin, respectively, and compared to WT controls (set to one arbitrary units, A.U.). Data represent mean ± SEM, $n = 4$ for each group, $^{*}p < 0.05$, Mann-Whitney test. **(B)** Dopamine levels in WT and *Rab39b* KO mouse midbrain were determined by ELISA for comparison. $n = 4$, $^{*}p < 0.05$, Mann-Whitney test.
